# Antiviral activity of natural phenolic compounds in complex at an allosteric site of SARS-CoV-2 papain-like protease

**DOI:** 10.1038/s42003-022-03737-7

**Published:** 2022-08-11

**Authors:** Vasundara Srinivasan, Hévila Brognaro, Prince R. Prabhu, Edmarcia Elisa de Souza, Sebastian Günther, Patrick Y. A. Reinke, Thomas J. Lane, Helen Ginn, Huijong Han, Wiebke Ewert, Janina Sprenger, Faisal H. M. Koua, Sven Falke, Nadine Werner, Hina Andaleeb, Najeeb Ullah, Bruno Alves Franca, Mengying Wang, Angélica Luana C. Barra, Markus Perbandt, Martin Schwinzer, Christina Schmidt, Lea Brings, Kristina Lorenzen, Robin Schubert, Rafael Rahal Guaragna Machado, Erika Donizette Candido, Danielle Bruna Leal Oliveira, Edison Luiz Durigon, Stephan Niebling, Angelica Struve Garcia, Oleksandr Yefanov, Julia Lieske, Luca Gelisio, Martin Domaracky, Philipp Middendorf, Michael Groessler, Fabian Trost, Marina Galchenkova, Aida Rahmani Mashhour, Sofiane Saouane, Johanna Hakanpää, Markus Wolf, Maria Garcia Alai, Dusan Turk, Arwen R. Pearson, Henry N. Chapman, Winfried Hinrichs, Carsten Wrenger, Alke Meents, Christian Betzel

**Affiliations:** 1 Department of Chemistry, Institute of Biochemistry and Molecular Biology, Laboratory for Structural Biology of Infection and Inflammation, Universität Hamburg, Build. 22a, c/o DESY, 22607 Hamburg, Germany; 2 Hamburg Centre for Ultrafast Imaging (CUI), Universität Hamburg, Luruper Chaussee 149, 22761 Hamburg, Germany; 3 Department of Parasitology, Institute of Biomedical Sciences at the University of São Paulo, São Paulo, Brazil; 4 Center for Free-Electron Laser Science, CFEL, Deutsches Elektronen Synchrotron DESY, Notkestrasse 85, 22607 Hamburg, Germany; 5 Diamond Light Source Ltd. Diamond House, Harwell Science and Innovation Campus, Didcot, OX11 0DE UK; 6 European XFEL GmbH. Holzkoppel 4, 22869 Schenefeld, Germany; 7 Department of Biochemistry, Bahauddin Zakariya University Multan-, 60800 Punjab, Pakistan; 8 Pólo TerRa, São Carlos Institute of Physics, University of São Paulo, São Carlos, Brazil; 9 Department of Microbiology, Institute of Biomedical Sciences at the University of São Paulo, São Paulo, Brazil; 10 Clinical Laboratory, Hospital Israelita Albert Einstein, São Paulo, Brazil; 11 Scientific Platform Pasteur USP, São Paulo, Brazil; 12 European Molecular Biology Laboratory Hamburg, c/o DESY, Notkestrasse 85, 22607 Hamburg, Germany; 13 Photon Science, Deutsches Elektronen Synchrotron (DESY), Notkestrasse 85, 22607 Hamburg, Germany; 14 Fraunhofer Institute for Translational Medicine and Pharmacology (ITMP), Schnackenburgallee114, 22525 Hamburg, Germany; 15 Department of Biochemistry & Molecular & Structural Biology, Jozef Stefan Institute, Jamova 39, 1 000 Ljubljana, Slovenia; 16 Centre of excellence for Integrated Approaches in Chemistry and Biology of Proteins (CIPKEBIP), Jamova 39, 1 000 Ljubljana, Slovenia; 17 Institut für Nanostruktur- und Festkörperphysik, Universität Hamburg, Luruper Chaussee 149, 22761 Hamburg, Germany; 18 Department of Physics, Universität Hamburg, Luruper Chaussee 149, 22761 Hamburg, Germany; 19 Institute of Biochemistry, Universität Greifswald, Felix-Hausdorff-Str. 4, 17489 Greifswald, Germany

**Keywords:** Structural biology, Biochemistry

## Abstract

SARS-CoV-2 papain-like protease (PLpro) covers multiple functions. Beside the cysteine-protease activity, facilitating cleavage of the viral polypeptide chain, PLpro has the additional and vital function of removing ubiquitin and ISG15 (Interferon-stimulated gene 15) from host-cell proteins to support coronaviruses in evading the host’s innate immune responses. We identified three phenolic compounds bound to PLpro, preventing essential molecular interactions to ISG15 by screening a natural compound library. The compounds identified by X-ray screening and complexed to PLpro demonstrate clear inhibition of PLpro in a deISGylation activity assay. Two compounds exhibit distinct antiviral activity in Vero cell line assays and one inhibited a cytopathic effect in non-cytotoxic concentration ranges. In the context of increasing PLpro mutations in the evolving new variants of SARS-CoV-2, the natural compounds we identified may also reinstate the antiviral immune response processes of the host that are down-regulated in COVID-19 infections.

## Abstract (web summary)

Three natural phenolic compounds are found to bind to an allosteric site in SARS-CoV-2 papain-like protease and exhibit antiviral activity in vitro, showing potential as starting scaffolds for drug design.

## Introduction

The coronavirus disease COVID-19, caused by SARS-CoV-2, remains devastating with high numbers of infections and deaths^1^. Several approved and highly effective vaccines against COVID-19 were developed worldwide in only a year. However, these vaccines are not uniformly available around the world, and consequently new SARS-CoV-2 variants have already emerged which may impact the effectiveness of the available vaccines in the future^2^. In parallel, more efforts are needed to identify and optimize alternative treatments for patients infected by SARS-CoV-2, who do not respond to or cannot tolerate vaccines^3^. Hence, research is ongoing at a rapid pace to identify effective drug candidates by applying complementary strategies. One approach, we followed recently, is the massive X-ray crystallographic screening for inhibitors of SARS-CoV-2 main protease Mpro, an essential protein in the viral replication process and hence an important drug target^4^. We identified six compounds inhibiting Mpro that showed antiviral activity and these compounds are currently approaching the step of pre-clinical investigations^4^.

The positive-sense single-stranded RNA genome of coronaviruses encodes 16 nonstructural polyproteins (nsps 1–16). The Papain-like protease (PLpro) is a domain that is part of the nsp3 gene, the largest mature SARS-CoV-2 protein^5^. PLpro is required to recognize and cleave the motif LXGG within preprocessed polyproteins between nsp1/2, nsp2/3 and nsp3/4 into functional units for initiation, replication, and transcription of the viral genome^6,7^. Apart from the proteolytic activity, PLpro can also bind and cleave ubiquitin chains or ISG15 (interferon-stimulated gene product 15) from ubiquitinated or ISGylated proteins^8^. The deubiquitinase (DUB), as well as deISGylating activities are vital for the coronaviruses to antagonize the host immune responses. It has been shown that mono-ubiquitination at the endoplasmic reticulum (ER) membranes regulate endocytosis, and vesicle trafficking and thus is important for coronavirus propagation^9^. On the other hand, ISG15 causes metabolic pathway modifications towards excessive inflammatory and autoimmune responses due to interferon system dysregulations^10,11^. Target proteins for the coronavirus, such as IRF3 (Interferon regulatory factor 3) need to conjugate with ISG15 to be correctly phosphorylated for their entry into nucleus where they are required for downstream signaling events for e.g., via the IFN-β pathway^12^ to elicit an antiviral immune response. Therefore, the cysteine protease activity, together with the deubiquitinase and deISGylating activity of PLpro undoubtedly makes this enzyme a very promising target for drug discovery investigations^13,14^. Furthermore, the importance of PLpro as a drug target was highlighted in several recent studies^13–22^ that have identified novel and unexpected mutations in the PLpro domain of the nsp3 gene from the SARS-CoV-2 variants of concern (VOC), currently circulating in different parts of the world^23,24^.

The crystal structure of SARS-CoV PLpro, in complex with Lys48-linked di-ubiquitin^25^ (PDB code 5E6J), clearly evokes a mechanism of DUB action in which the LXGG residues at the C-terminal of the ubiquitin molecule bind in a cleft (Ub S1 proximal binding site) located close to the catalytic active site, allowing efficient cleavage. In addition, the recently obtained crystal structure of SARS-CoV-2 PLpro, in complex with mouse-ISG15^26^, demonstrates that the N-terminal region of ISG15 interacts with PLpro at a binding site termed as ISG15/Ub S2 distal binding site. Compared to the crystal structure of ISG15 (PDB code 5TLA), the N-terminal part of the ISG15 molecule in the complex structure of SARS-CoV-2 PLpro is rotated by about 90^°^ and coordinates with the S2-helix. These different binding events explain and highlight why SARS-CoV-2 PLpro and SARS-CoV PLpro, despite sharing 83% sequence identity, show different substrate preferences. Recently data were published showing higher affinity and specificity of SARS-CoV-2 PLpro to ISG15, whereas SARS-CoV PLpro preferentially cleaves ubiquitin chains, which may be associated to the substantial higher morbidity and mortality of SARS-CoV-2 in comparison to SARS-CoV infections^27^.

Until now several high throughput assay screening (HTS) activities, focussed on identifying potential inhibitors of PLpro using repurposing compound libraries were initiated. Unfortunately, these were not very successful in obtaining hits of compounds that could be further developed to obtain an effective antiviral drug^27^. Hence, we set out a different strategy to explore a unique library consisting of 500 natural compounds assembled and characterized by the Molecular Bank, ICCBS, Karachi, Pakistan, by structure-based drug design (https://iccs.edu/page-mol-bank). Natural compounds of the ICCBS Molecular Bank extracted from plants present characteristics such as high chemical diversity, medically relevant anti-tumor, anti-oxidant, anti-inflammatory and importantly antiviral action with typically milder or no side effects, in addition to lower cost of production as compared to most available drugs on the market^28^. A number of these compounds also have a long history of use as drug molecules to treat distinct human diseases, including viral infections, such as Hepatitis C virus infection^29^. Recent reports also demonstrate the potential use of plant molecules and their secondary metabolites against SARS-CoV-2, and other human coronaviruses^30–32^ by applying in vitro and in silico approaches. However, to our knowledge, a systematic screening of natural products by structure-based drug discovery providing direct experimental data about complex formation was not available to date.

Our high throughput screening by X-ray crystallography identified three natural compounds bound to PLpro. All three compounds, 4-(2-hydroxyethyl)phenol (YRL), 4-hydroxybenzaldehyde (HBA), and methyl 3, 4-dihydroxybenzoate (HE9) are phenol derivatives, classified as polyphenols, an important and major class of bioactive compounds present in plants. This vast group of bioactive compounds is divided into five major classes: hydroxybenzoic acids, hydroxycinnamic acids, flavonoids, stilbenes, and lignans. In addition to the well-known anti-oxidant and anti-inflammatory activities of phenol derivatives, several studies have reported their anti-viral potential against Epstein-Barr virus^33,34^, enterovirus 71^35^, herpes simplex virus (HSV)^36^, influenza virus^37^, and other viruses causing respiratory tract-related infections^38^.

Interestingly, all three compounds YRL, HBA and HE9 bind at the same, and yet unexplored ISG15/Ub-S2 allosteric binding site in the thumb region of PLpro by forming specific interactions and clearly inhibit PLpro in deISGylation activity assays. None of these lead compounds are cytotoxic in cellular cytotoxicity assays and therefore could be promising lead compounds with the potential to be developed as specific coronaviral PLpro inhibitors. Significantly, two of them exhibit antiviral activity and one inhibits cytopathic effects in the range of 60–80% in a non-cytotoxic concentration range up to 100 µM in cellular assays. These three natural phenolic compounds undoubtedly provide a scaffold as antiviral drugs for further development and optimization towards the prevention and/or reduction of SARS-CoV-2 viral replication, and to reinstate and support the innate immune response of the host in parallel.

## Results and discussion

### X-ray screening of a natural compound library identifies three allosteric inhibitors of SARS-CoV-2 PLpro

We initiated a structure-based drug discovery approach to identify potential inhibitors for PLpro by X-ray screening of 500 compounds from the ICCBS Molecular Bank. SARS-CoV-2 PLpro was expressed recombinantly in *Escherichia coli*, and purified to homogeneity as a monomer (see Methods). Wild-type enzyme crystals were obtained in a stable and reproducible condition and diffracted X-rays to a high resolution of 1.42 Å. Data collection and refinement statistics are summarized in Table 1. The electron density maps obtained for the wild-type enzyme allowed the elucidation of all 315 amino acid residues, the zinc ion, and 529 solvent water molecules. Further, a glycerol molecule from the cryoprotectant, used for freezing crystals, could be modeled in the electron density map, as well as a phosphate and two chloride ions.

**Table 1:** Data collection and refinement statistics.

PDB code/in complex with	7NFV/native	7OFS/YRL	7OFU/HE9	7OFT/HBA
Resolution range (Å)	48.89–1.42 (1.47–1.42)	44.78–1.90 (1.97–1.90)	48.87–1.72 (1.78–1.72)	40.81–1.95 (2.02–1.95)
Space group	P 3_2_ 2 1	P 3_2_ 2 1	P 3_2_ 2 1	P 3_2_ 2 1
Unit cell a, b, c (Å) α,β,γ (°)	82.33, 82.33, 134.32 90, 90, 120	82.40, 82.40, 134.33 90, 90, 120	82.39, 82.39, 134.14 90, 90, 120	81.61, 81.61, 134.37 90, 90, 120
Total number of reflections	5,275,155 (383,126)	465,619 (43,757)	624,464 (631,78)	416,072 (398,83)
Unique number of reflections	99,791 (9853)	42,250 (4154)	565,58 (5573)	38,419 (3775)
Multiplicity	52.9 (38.9)	11.0 (10.5)	11.0 (11.3)	10.8 (10.6)
Completeness (%)	99.94 (99.53)	99.92 (99.90)	99.95 (99.82)	99.95 (99.87)
Mean I/sigma(I)	29.17 (0.68)	15.25 (0.86)	19.44 (0.87)	20.61 (1.31)
R-merge	0.07248 (6.281)	0.08749 (2.58)	0.07159 (2.692)	0.08102 (1.916)
CC_1/2_	1 (0.418)	0.999 (0.505)	0.999 (0.453)	0.999 (0.667)
**Refinement**				
Reflections used	99,737 (9810)	42,226 (4150)	56,587 (5565)	38,403 (3766)
Reflections used for R-free	5022 (474)	2036 (187)	2834 (259)	1931 (187)
R-work	0.154 (0.330)	0.185 (0.344)	0.175 (0.341)	0.181 (0.289)
R-free	0.171 (0.350)	0.214 (0.368)	0.202 (0.386)	0.213 (0.356)
Protein atoms	2674	2548	2599	2545
Ligand atoms	0	26	46	20
Solvent atoms	550	205	328	270
RMS (bonds) Å	0.020	0.009	0.016	0.008
RMS (angles)°	2.30	1.23	1.933	1.23
Ramachandran favored (%)	96.49	97.44	96.45	97.12
Ramachandran allowed (%)	3.51	2.24	3.19	2.88
Ramachandran outliers (%)	0.00	0.32	0.35	0.00

**Fig. 1:**
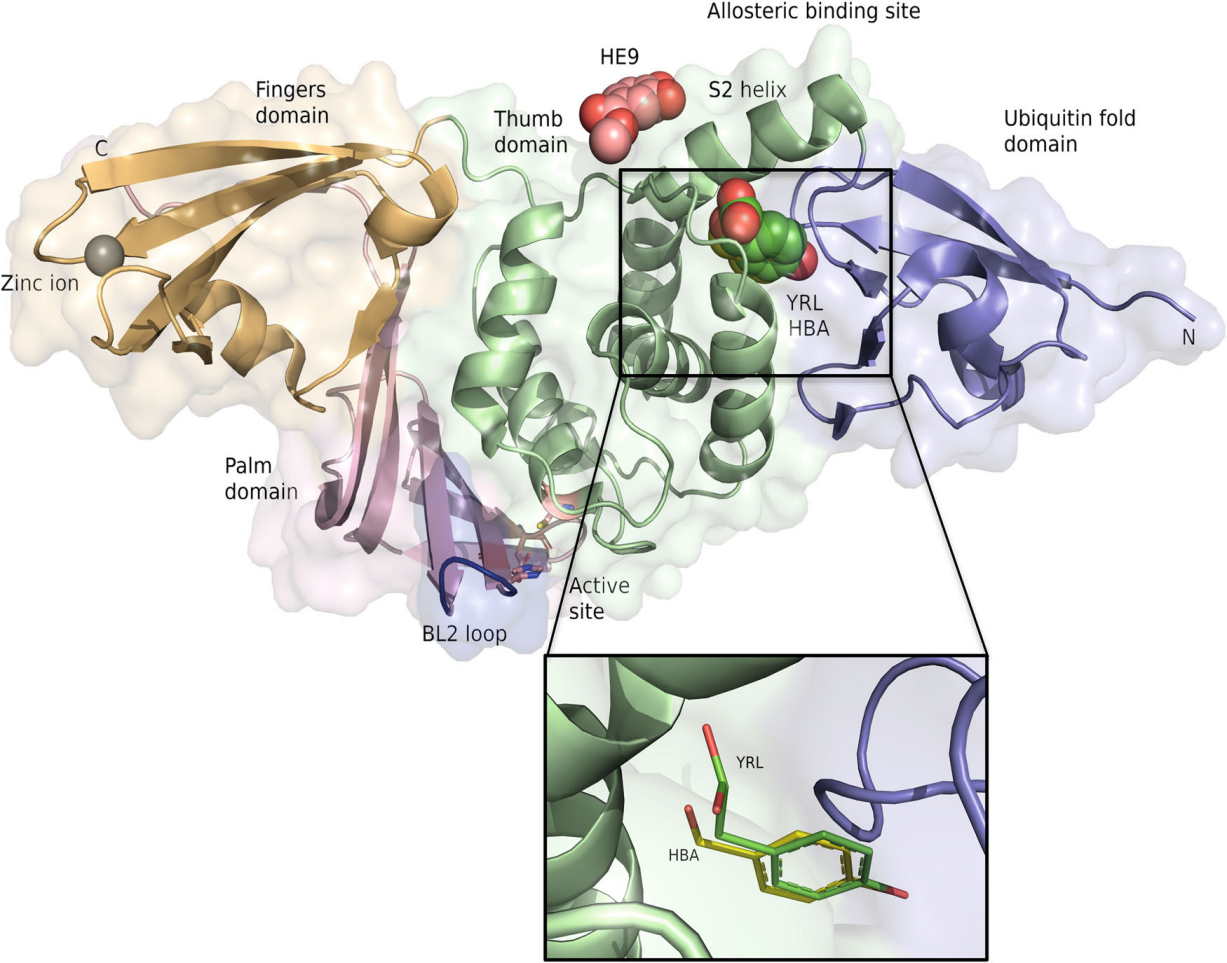
Crystal structures of SARS-CoV-2 PLpro complexes with the three natural compounds. PLpro domains are depicted in a right-handed architecture, ubiquitin-fold like (blue), thumb (green), palm (salmon pink), and fingers (light orange). Catalytic active site residues Cys 111, His 272, and Asp 286 are represented as sticks and a zinc ion in the fingers domain is shown as a gray sphere. The flexible blocking loop (BL2 loop) that changes conformation in the context of substrate binding is shown in blue. YRL (green spheres), HBA (yellow spheres) and HE9 (pink spheres) compounds bind at the allosteric site that is located about 30 Å apart to the active site. S2 helix involved in the interaction of the ISG15 molecule is indicated. The inset shows an enlarged view of the two compounds HBA and YRL in the binding site.

PLpro folds with a right-handed architecture consisting of thumb, palm, and fingers domains with a catalytic triad consisting of Cys111-His272-Asp286 and a N-terminal ubiquitin-like domain (Fig. 1). Four cysteine side chains coordinate a zinc ion, constituting a ‘zinc finger motif’ that is essential for structural stability and protease activity of the enzyme^39^. The overall structure of SARS-CoV-2 PLpro is homologous to SARS-CoV PLpro (PDB code 2FE8) that shares a sequence identity of 83% with a r.m.s.d. of 0.58 Å for 260 equivalent Cα atoms and also to MERS-CoV PLpro (PDB code 4RNA) despite a lower sequence identity of 29% and a corresponding r.m.s.d. of 1.83 Å for 258 equivalent Cα atoms (Figs. S4 and S8). The most structurally dynamic regions are the ubiquitin-fold like, and the zinc fingers domains. The catalytic active site region is conformationally well conserved among the different coronaviral PLpro enzymes. The access to the active site is regulated via a flexible loop named “blocking loop 2” (BL2, Fig. 1), as this loop changes from an ‘open’ to a ‘closed’ conformation in the context of substrate binding^40^. A number of known PLpro inhibitors bind at this site, including the high affinity inhibitor GRL0617, and structural variations have been observed in this loop among different PLpro enzymes^41^.

Crystals of SARS-CoV-2 PLpro, in complex with the three natural compounds, were obtained by co-crystallization using the vapor diffusion method in a screening approach utilizing 500 molecules from a library of natural compounds. Crystals were grown in the same condition as for the native PLpro and diffraction datasets were collected in the resolution range of 1.7–1.9 Å. Over 2000 crystals were harvested, and multiple datasets for each compound were collected that resulted in ~2500 diffraction datasets (Fig. S12). PLpro structures complexed with inhibitor compounds were solved using the ligand-free PLpro (PDB code 7NFV) as reference model for consistent indexing of datasets with a previously established automatic pipeline^4^ (see Methods). Data collection and refinement statistics are summarized in Table 1. The three complex structures obtained superimpose well with the ligand-free structure 7NFV with a r.m.s.d. of 0.26 Å (298 Cα atoms) to 7OFS, r.m.s.d. of 0.07 Å (283 Cα atoms) to 7OFU and r.m.s.d. of 0.33 Å (299 Cα atoms) to 7OFT, respectively. The three compounds bind at the ISG15/Ub S2 allosteric site, near the thumb region of PLpro (Fig. 1) which is located about 30 Å apart to the active site residue Cys 111. The interaction between these allosteric inhibitors and PLpro are formed via hydrogen bonds, hydrophobic, and π-stacking interactions (Figs. S5, S6, and S7).

### Molecular basis of inhibition by the three allosteric inhibitors of SARS-CoV-2 PLpro

The natural compound 4-(2-hydroxyethyl)phenol (YRL), isolated from *Lawsonia alba*, is a well-known antioxidant and an anti-arrythmia agent (https://pubchem.ncbi.nlm.nih.gov/compound/2-_4-Hydroxyphenyl_ethanol). YRL binds to PLpro at the ISG15/Ub S2 allosteric binding site in a hydrophobic cavity with a predicted binding energy of −7.17 kcal/mol (calculated using Prodigy^42^). The benzene core is covered by hydrophobic interactions with side chains of Val 11, Val 57, Pro 59, Tyr 72, and Leu 80. The main-chain nitrogen atom of Leu 80 is hydrogen bonded *via* a water molecule to the hydroxyethyl substituent of YRL. Interestingly, the hydroxyethyl substituent is observed with two alternative conformations and is refined to equal occupancy in the complex structure. One conformation forms a hydrogen bond to carbonyl backbone of Asp 76, the alternative hydroxyl to the carbonyl of Thr 74. Both alternative hydroxyl groups replace water molecules in the ligand-free enzyme and have contacts to solvent water molecules at the entry of the binding pocket. The phenolic hydroxyl is hydrogen bonded to carbonyl oxygen of Val 57 at the bottom of the binding pocket to complete the interaction of the ligand YRL in the PLpro-YRL complex structure (Fig. S5).

The second compound, 4-hydroxybenzaldehyde (HBA), isolated from *Acalypha torta*, is a well-known anti-tumor agent^43,44^. The calculated binding energy for the interaction of the HBA ligand to PLpro is −6.97 kcal/mol. The benzene core and the phenolic hydroxyl is observed in the same position and, thus, has similar interaction to PLpro as described above for YRL (Fig. S6). The distance of the phenolic hydroxyl of both compounds to the Cα of Val 11 indicates a C-H···O hydrogen bond (3.4 Å). The aldehyde substituent has weak water contacts at the entrance of the binding pocket. The remarkable structural change in PLpro to accommodate these two compounds is that the side chain of Leu 80 has to tilt away in the complex PLpro structures (Fig. S13).

The third compound, methyl 3,4-dihydroxybenzoate (HE9), isolated from *Tagetes patula* (marigold), is a major diphenol found in green tea with antioxidant and anti-inflammatory effects^45^ (https://pubchem.ncbi.nlm.nih.gov/compound/Methyl-3_4-dihydroxybenzoate). The calculated binding energy for this interaction is −6.15 kcal/mol. HE9 binds at the surface of PLpro adjacent to the binding cavity with ligands HBA and YRL. The interaction to PLpro is formed by hydrogen bonds of the dihydroxyphenol edge to the side chain of Glu 70. Hydrophobic interactions are observed including the π-stacking with the imidazole of His 73 and contacts of the benzene core to the side chain of Phe69 (Fig. S7). The extraction, isolation and purification of the three compounds HBA, YRL and HE9 are presented in supplementary notes 1, 2 and 3. The NMR spectra of HBA, YRL, and HE9 are shown in Supplementary Figs. S1, S2, and S3 respectively.

We determined the binding constants for the ligands HBA and HE9 using their quenching effect on the fluorescence for PLpro applying the nanoDSF (nano Differential Scanning Fluorimetry) method^46^. This resulted in *K*_d_ values of ~400 μM for HBA and 1 mM for HE9 (depending on the emission wavelength used, Fig. S15). YRL showed a high intrinsic fluorescence intensity and therefore could not be used in a fluorescence titration experiment. Since YRL is structurally very homologous to HBA and it binds in the same pocket of PLpro, we assume a similar binding affinity.

**Fig. 2:**
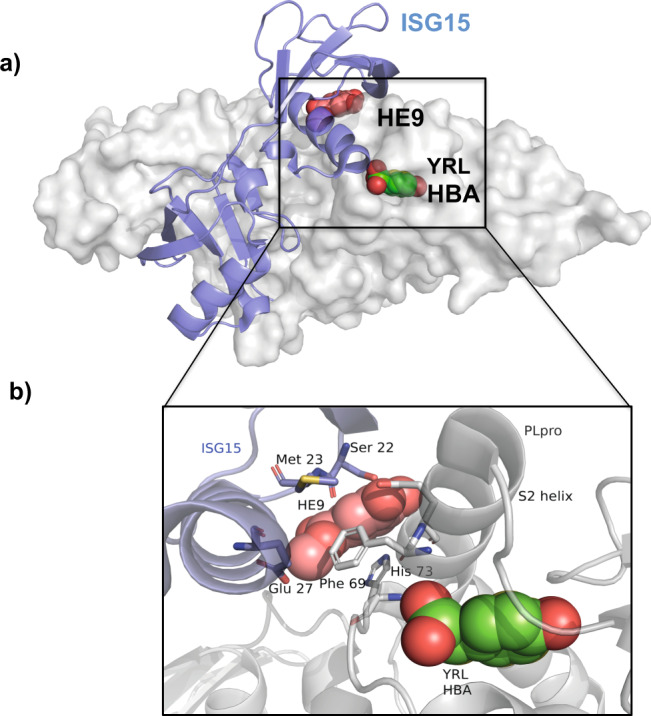
Interaction of the ISG15 molecule to PLpro is disrupted by the binding of the three natural compounds. **a** Superposition of the crystal structures of SARS-CoV-2 PLpro-C111S in complex with mouse-ISG15 (PDB code 6YVA, ISG15 molecule in blue) with SARS-CoV-2 PLpro+HE9 (PDB code 7OFU, in gray surface representation). The three compounds YRL, HBA, and HE9 are depicted as spheres. **b** Close-up view of the ISG15 binding site. ISG15 molecule is shown as a cartoon representation (blue) with the interacting residues Ser 22, Met 23, and Glu 27 in sticks. The bound inhibitor compounds (spheres) clearly prevent the binding of the ISG15 molecule to the S2 binding site of PLpro.

The described interaction networks of the three compounds involve amino acid residues Phe 69, Glu 70, and His 73 that have been previously shown to interact with ISG15 and Lys48di-Ub molecules^25,26^. Crystal structures of SARS-CoV PLpro, in complex with Lys48di-Ub (5E6J), and SARS-CoV-2 PLpro in complex with mouse-ISG15 (6YVA) supported by molecular dynamics simulations clearly reveal the hydrophobic interactions between these amino acid residues in PLpro with either ISG15 or Lys48-di ubiquitin molecules^26^. A superimposition of the PLpro+inhibitor complex structures with PLpro+ISG15 complex (Fig. 2a), shows that the binding of the natural compounds clearly disrupts and prevents the binding of ISG15 to PLpro. Critical residues Ser 22, Met 23, and Glu 27 located in the binding surface of ISG15 are no longer available to form interactions with PLpro (Fig. 2b) upon binding of these natural products.

PLpro enzymes share the same core residue, SARS-CoV Phe 70 and SARS-CoV-2 Phe 69 at the ISG15 binding site. A mutation of this residue in PLpro to alanine decreased the enzymatic activity, and also resulted in a slower reaction with ISG15, as compared to the wild-type enzyme^26^. In MERS-CoV PLpro, Phe 69 is replaced by a lysine residue (F69K) and His 73 by a glycine residue (H73G). These variations might account for the different substrate preferences among SARS-CoV and SARS-CoV-2 and MERS PLpro. It can be seen from a superimposition of the crystal structure of the MERS-CoV PLpro+ISG15 (6BI8), with the SARS-CoV-2 PLpro-HE9 complex structure (7OFU), that F69K and H73G substitutions confer different surface properties for the interaction with ISG15 (Fig. S10a, b).

Cleavage of polyubiquitin chains by SARS-CoV-2 PLpro is significantly enhanced when a longer ubiquitin chain is used. This demonstrates that either Ub or ISG15 molecules bind not only to the Ub-S1 binding site but also to the Ub-S2 site, facilitated by the conserved S2 helix in PLpro, being important for the enzymatic activity^47^. Superimposition of crystal structures of PLpro in complex with Lys48 linked di-ubiquitin (5E6J) with the PLpro-HE9 complex (7OFU) showed that key residues involved in ubiquitination, Lys 11 and Lys 48 in the S2-Ub binding site, are no longer available for binding either to ubiquitin or ISG15 (Fig. S9 a, b). Hence, a clear molecular basis for the inhibition emerges from the three PLpro inhibitor complex structures, showing that PLpro-ISG15 interactions are affected upon the binding of the three phenolic natural products.

### In vitro enzymatic assays to monitor the inhibition of SARS-CoV-2 PLpro

Fluorescence activity assays were carried out to assess the inhibitory effect of the three compounds (HE9, YRL, and HBA), co-crystallized with the wild-type SARS-CoV-2 PLpro. A catalytically inactive PLpro mutant (C111S) was used as a control applying ISG15-Rhodamine and Ub-Rhodamine as substrates. Wild type PLpro (WT PLpro) at 10 nM concentration represents 100% of deISGylation activity and the three natural compounds YRL, HBA, and HE9 at 50 µM show clear inhibition of PLpro enzymatic activity using ISG15-Rhodamine as the substrate. In particular the two compounds, HBA and YRL significantly decreased PLpro activity by ~73 and 70% respecitively, followed by HE9 inhibiting to ~55% in a deISGylation assay (Fig. S11); while the inhibition was not as pronounced applying Ub-Rhodamine as substrate (Fig. S16). It can be rationalized that the binding of the natural compounds at the S2 helix region clearly prevents the essential PLpro/ISG15 molecular interactions required for the deISGylation mechanism of PLpro.

**Fig. 3:**
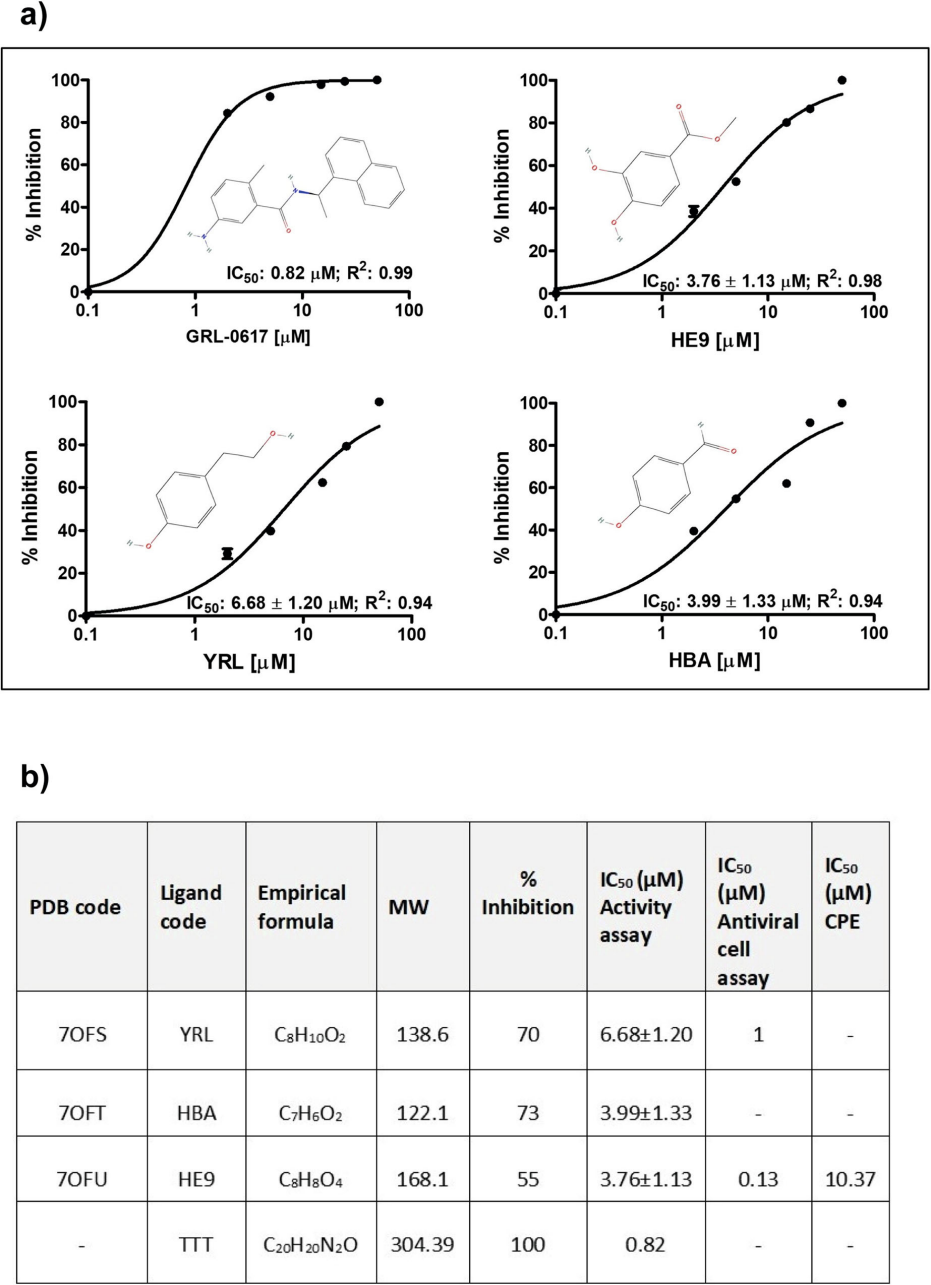
Inhibition of PLpro by the three natural compounds in deISGylation assay with ISG15-Rh substrate. **a** IC_50_ determination was performed with ISG15-Rhodamine as the substrate at a concentration of 250 nM. A gradient concentration of all three compounds YRL, HBA, HE9, and the inhibitor GRL-0617 as a control in the range from 2 to 50 µM was used in the reaction mixture. IC_50_ values were calculated by fitting the data to a sigmoidal dose-response-inhibition function and are presented in the log scale for interpolation. Individual data points represent the mean of normalized relative fluorescence unit per min ±SD from triplicates. **b** Summary of the inhibition profiles for the three natural compounds YRL, HBA, HE9, and the control compound GRL-0617 (TTT) obtained from enzyme activity assays and cell line antiviral assays.

Further, we determined the inhibition efficacy by performing in vitro IC50 assays, which demonstrated efficient inhibition of the three compounds namely HE9 (methyl 3,4-dihydroxybenzoate), HBA (p-hydroxybenzaldehyde) and YRL (4-(2-hydroxyethyl)phenol), in a concentration range of 3.76 ± 1.13, 3.99 ± 1.33 and 6.68 ± 1.20 μM respectively. The compound, GRL0617 (5-Amino-2-methyl-N-[(1R)-1-(1-naphthalenyl) ethyl] benzamide), a known inhibitor of PLpro was used as a control (Fig. 3a). To characterize the specificity of the three compounds towards PLpro, enzymatic inhibition assays with the SARS CoV-2 main protease (Mpro) and applying the three compounds were also performed, considering the same PLpro protocol with incubation time up to 6 h. Results clearly demonstrated no inhibitory effect of Mpro activity in the presence of the three natural compounds when compared to the known Mpro inhibitor GC-376, as shown in Fig. S14. Antiviral activities for these natural phenolic compounds, either in crude or purified form, were reported previously^35,38^ and can now be related to PLpro inhibition, as shown in our activity assays. However, we cannot exclude the interaction of these compounds with other vital cellular target proteins. Further, it has recently been demonstrated that the PLpro minimal domain is unable to cleave the Nsp1/2 fusion protein and it has been demonstrated that the full-length Nsp3 core protein is required to represent the PLpro peptidase activity, which needs to be considered in terms of drug discovery investigations^48^.

### Cellular assays to monitor the inhibition of SARS-Co-V-2 PLpro

**Fig. 4:**
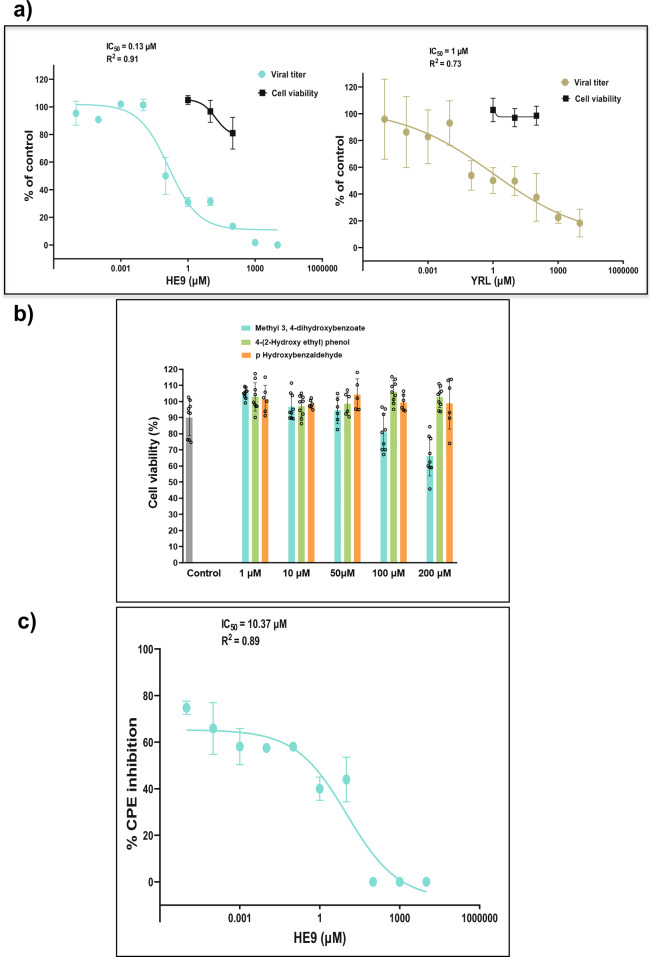
Effect of the natural compounds on SARS-CoV-2 loading in Vero cells. **a** The viral titer and cell viability were quantified by qRT-PCR (●) and CellTiter-Glo luminescence method (■), respectively. IC_50_- and R-squared values for viral titers are shown. IC_50_-values were calculated by fitting the data to the sigmoidal function as previously described^4^. Compounds concentrations are presented in log scale for interpolation. HE9 was diluted to a stock concentration of 100 mM in DMSO, while YRL was diluted in sterile water to a 50 mM stock concentration. All compounds were stored at −20 °C. Individual data points represent means ± SD from four independent replicates in two biological experiments. Values were plotted in a line graph with error bars displaying standard deviation. **b** Cell viability in the presence of the three compounds was determined by CellTiter-Glo luminescence method. Individual data points from three independent replicates in three biological experiments. **c** CPE inhibition was determined by CellTiter-Glo luminescence method. IC_50_- and R-squared values are shown. IC_50_-values were calculated by fitting the data to the sigmoidal function. Individual data points represent means ± SD from three independent replicates in one biological experiment. Values were plotted in a line graph with error bars displaying standard deviation.

Considering the observed inhibitory synergic effect, combined with the molecular regulative antiviral homeostasis function of ISG15 in the human host, we investigated the inhibitory efficacy of the compounds HE9, HBA, and YRL towards viral replication and the cytopathic effect in living cells using Vero cell line assays. Two distinct approaches were applied, qRT-PCR reaction as previously described^4,25^ and CellTiter-Glo assay, a luciferase reporter assay to determine the ATP level present in viable cells^49^. Screening experiments started at 5 mM of the three compounds and used a 10-point, 1:10 dilution series with infections being performed at a multiplicity of infection (MOI) value of 0.01. The two compounds HE9 and YRL showed a reduction of viral RNA (vRNA) replication with IC_50_ values of 0.13 and 1 µM respectively, with no associated cell toxicity at 100 µM (Fig. 4a). Cell viability experiments were performed simultaneously under the same conditions in the absence of virus and revealed no effects on cell viability at concentrations where the compounds showed antiviral activity (Fig. 4b).

The compound HE9 significantly reduced viral RNA (vRNA) replication among the three compounds studied and was further evaluated to determine the effective concentrations that can reduce not only vRNA levels but also SARS-CoV-2 virus infectious particles applying a cytopathic effect (CPE) inhibition assay (Fig. 4c). An active compound was the one which exhibited a CPE inhibition of >50% without compromising cell viability. We were unable to fit a sigmoidal curve to the data for the compounds HBA and YRL. Importantly, treatment of the cells with the compound HE9 reduced the viral replication and showed an ability to inhibit CPE with IC_50_ = 10 µM (Fig. 4c). These results from the cellular assays are in line with the in vitro enzymatic studies using deISGlyation assays and clearly demonstrate that the compound HE9 is a potential inhibitor of PLpro, which can protect the host cells from the viral CPE.

PLpro from SARS-CoV, MERS-CoV and other coronaviruses are able to inactivate the components of type I interferon partly mediated by their deubiquitination and deISGylation functions^5,25^. A unique feature of Vero cells is that they are interferon-deficient lacking the production of interferons type I (IFN), the antiviral signaling proteins typically produced by mammalian cells^50^ and known to strongly express ISG15. Thereby, the cellular viability, the inhibition of viral replication in a micromolar range and the effective inhibition of the cytopathic effect in the presence of HE9 in Vero cells were modulated via an alternative cellular pathway during the infection by SARS-CoV-2.

Recent studies utilizing similar cell-based protocols to assess the replication of SARS-CoV-2 with the Vero cell line have reported a stable viral replication curve between 24 and 40 h post infection, followed by an evident declining of replication and a strong cytopathic effect on the cellular viability^51^. This observation was not seen in our study when the HE9 compound was titrated in the same Vero cells line. In the same study, a significant inhibition of the viral replication was detected when Vero cells were infected with SARS-CoV-2 and treated independently with IFN-β1a and IFN-α (type I IFN). Moreover, treatment of un-infected Vero cells with human interferon type III (IFN-λ1) stimulated endogenous cellular expression of other ISGs, such as MxA (myxovirus resistance protein), PKR (protein kinase R), OAS-1 (2′,5′-oligoadenylate synthetase), SOCS-1 (Suppressor Of Cytokine Signaling 1) and Rig-1 (Retinoic acid-inducible gene I)^52,53^, which suggest that Vero cells, even devoid of type I IFNs, can elicit functional IFN III responses^54^. Thus, the compound HE9 could indirectly attenuate the viral activity by reversing the host deubiquitination events linked to the IFN response deficiency in Vero cells.

## Conclusion

We have identified three phenolic compounds that inhibit PLpro by binding at an allosteric S2 site, an interaction and binding region for the ISG15 molecule. All three compounds show inhibition to PLpro in a deISGylation assay and demonstrate no inhibition in an enzymatic activity assay performed with the main protease, Mpro. Interestingly, two compounds exhibit distinct antiviral activity in Vero cell line assays and one compound additionally inhibited a cytopathic effect in non-cytotoxic concentration ranges. The binding affinities for the three compounds are in the lower micromolar range, indicating the compounds are weak binders to PLpro, but certainly provide valuable starting scaffolds as lead compounds targeting an allosteric binding site in PLpro. Molecular docking studies with the three phenolic compounds either covalently linked or extended with the thiosemicarbazone structures exhibit an increase of predicted binding energies by 0.8–1.8 kcal/mol in comparison to the unextended initial compounds^55^. Thus, the observed binding affinities and specificities of the three compounds can be improved by a systematic SAR (structure-activity relationship) analysis.

In summary, the high-resolution PLpro complex structures with phenolic natural compounds YRL, HBA, and HE9 complemented by enzymatic and cellular assays, provided a molecular basis to understand the inhibitory mechanism, a route to develop effective PLpro inhibitors of substrates binding to the PLpro S2 helix binding pocket and shed light on the mode of ISGylation of COVID-19 viral proteins as a new approach for preventing their interaction with human host cellular pathways. We believe that this approach to inhibit PLpro may hinder and reduce the viral ability to perform deISGylation in post COVID-19 viral complications, as well as providing more ISG15 within the lung tissues for the modulation of cytokine/chemokine production, to support the repair of the respiratory epithelium within COVID-19 infections^11^.

## Methods

### Cloning, protein overexpression, and purification of SARS-CoV-2 PLpro

A fragment of SARS-CoV-2 ORF pp1a/ab encoding the PLpro domain and corresponding to amino acids 746–1060 of non-structural protein 3 (YP_009742610.1) was cloned into pETM11(EMBL), which encodes N-terminal hexa-his tag followed by a tobacco etch virus (TEV) protease cleavage site. After cleavage by TEV protease extra two amino acids (GA) are left on the N-terminal of PLpro construct. The plasmid encoding the desired construct was transformed into *E. coli* Rosetta (DE3) cells (Merck, Germany) to perform expression via autoinduction medium, essentially as described before^56^ and using kanamycin for selection. An overnight cell culture was diluted and incubated in autoinduction medium containing 0.5 g L^−1^ β-D-glucose and 2 g L^−1^ lactose under constant shaking for 4 h at 37 °C and then in the presence of 100 µM ZnCl_2_ additionally over-night at 18 °C. Subsequently, cells were harvested by centrifugation and disrupted by sonication in lysis buffer (50 mM NaH_2_PO_4_, 150 mM NaCl and 10 mM imidazole, pH 7.2).

Cell extracts were maintained at 4 °C and centrifuged at 12,000 × *g* for 1 h. The clear supernatant was incubated with Ni-NTA affinity resin (Thermo Fisher Scientific, USA). PLpro was eluted by gravity flow using lysis buffer supplemented with 300 mM imidazole and subsequently incubated with TEV protease at a molar ratio of 20:1 in the presence of 1 mM DTT. Cleavage was performed during dialysis against 50 mM Tris, 150 mM NaCl and 1 mM DTT adjusted to pH 7.3 and for 14 h at 8 °C. After removing protease and the cleaved off tag by affinity chromatography, PLpro was purified to homogeneity using size-exclusion chromatography, i.e. a HiLoad 16/600 Superdex 75 column connected to an ÄKTA purifier (GE Healthcare, GB) equilibrated with 50 mM Tris, 150 mM NaCl and 1 mM TCEP at pH 7.5. Purity and integrity of the protein were verified via SDS polyacrylamide gel electrophoresis and DLS (Dynamic Light Scattering). The concentration of PLpro with a calculated molecular weight of 35,760 Da (ε_calculated_ = 45,270 M^−1^ cm^−1^) was adjusted to 20 mg mL^−1^ in preparation for vapor diffusion crystallization trials.

### Crystallization of ligand free SARS-CoV-2 PLpro and complexes with compounds

Initial crystallization screening experiments were performed using the sitting-drop vapor diffusion method utilizing the Oryx4 robot (Douglas Instruments) with the SWISSCI 96-well plates. Wizard™ Classic 1, 2, 3 and 4, JCSG+, PACT crystallization formulations were tried for initial screening experiments. Crystallization was performed with a protein:reservoir ratio of 2:1 at 4 and 20 °C. Initial hits were obtained from the Wizard screen, condition G11 (0.1 M acetate buffer pH 4.5, 0.8 M NaH_2_PO4/1.2 M K_2_HPO_4_) at 4 °C. Further optimization was done by changing the buffer to 0.1 M Tris-HCl pH = 8.0 and including 10% glycerol that resulted in 0.2–0.3 mm bipyramidal crystals. These crystals diffracted X-rays to a resolution of 1.42 Å.

PLpro complex crystals with compounds were grown by the co-crystallization method using the same condition as summarized above. 100 nL droplets of 10 mM compound solutions in DMSO from the Sadia Molecular Bank, Karachi library of natural compounds were applied onto a 96-well SWISSCI plate and the compounds were dried in vacuum before the addition of 200 nL of (20 mg/mL) PLpro protein solution and 100  nL of the crystallization condition (0.1 M Tris-HCl buffer pH 8.0, 0.8 M NaH_2_PO_4_/1.2 M K_2_HPO_4_ and 10% glycerol). The drops were equilibrated in a sitting drop vapor diffusion setup with 80 μl of reservoir solution. The plates were incubated at 4 °C and crystals appeared in 2 days and grew reproducibly to dimensions of approx. 0.2 × 0.3 × 0.2 mm^3^ in 4 days. Crystals were manually harvested directly from the drop and flash-frozen in liquid nitrogen for diffraction data collection.

### Data collection, structure solution, and refinement

Diffraction data from the ligand-free and complex PLpro crystals were collected at beamline P11, PETRA III/DESY, Hamburg. All datasets of ligand-free PLpro were processed using the program XDS^57^ with a reference dataset to ensure consistent indexing. From a total of 64 complete datasets, the strongest were selected based on (I/σ)^asymptotic^ greater than 20^58^. These 25 datasets were then subjected to iterative merging using CODGAS^59^ run with standard parameters. The best merged datasets was further manually filtered leading to the final dataset that contained five datasets. These were scaled with XSCALE^57^ and final merging and resolution cut-off was applied using AIMLESS^60^. Structure solution was achieved by molecular replacement method with PHASER^61^ using the PLpro coordinates with PDB code 7JRN as search model. Successive rounds of manual building with the program COOT^62^ and refinement with PHENIX^63^, the addition of phosphate, glycerol, chloride ions, and water solvent molecules to the model, followed by a final round of TLS refinement completed the structure refinement at a resolution of 1.42 Å.

An automatic data processing pipeline, hit finding, clustering^64^, PanDDA analysis^65^ and refinement protocols as described previously^4^ were used for the structure solution and analysis of PLpro in complex with the natural compounds from the library consisting of 500 compounds. Data processing with XDS resulted in 1469 datasets and includes more than one dataset per compound. Data quality indicators CC_1/2_ and Wilson B-factors are plotted as shown in Fig. S7. Final rounds of manual refinement with either Refmac^66^ or Phenix^67^ together with manual model building applying COOT resulted in the final refined structures. Data collection and refinement statistics for PLpro and complexes are summarized in Table 1, supplementary information. All figures were prepared using PyMol^68^

### SARS-CoV-2 PLpro inhibition assays and IC_50_ determination

Activity assays were performed for SARS-CoV-2 PLpro native and mutant enzyme (PLpro C111S mutant) to determine the deISGylation and deubiquitination activities effected by the three natural compounds, following previously published protocols^26,27,47^. The assays were performed with a total reaction volume of 100 µL in non-binding, black bottom, 96-well plate, and reactions were measured on a Tecan Infinite M plus plate reader (Tecan Group Ltd, Switzerland) using optical settings specific for ISG15-Rhodamine (UbiQ-127, UbiQ Bio) and Ubiquitin-Rhodamine (UbiQ-126, UbiQ Bio). ISG15-Rhodamine and Ub-Rhodamine are fluorogenic substrates that contain the cleavage sequence RLRGG recognized by PLpro at the C-terminus. The cleavage of the amide bond between the terminal glycine residue and the rhodamine110 fluorophore releases the fluorescent Rh110-morpholinecarbonyl that results in an increase of fluorescence intensity, measured as RFU (Relative Fluorescence Unit). The fluorophore has an excitation and emission at 492 and 525 nm respectively. The ISG15 substrate (UbiQ-127) and Ub substrate (UbiQ-126) were used at a final concentration of 100 nM and the concentration of PLpro was 10 nM in the assay. Relevant substrate and positive controls (GRL0617) was used throughout the assay. SARS-CoV-2 PLpro native, mutant, and substrates were diluted in assay buffer (20 mM Tris-HCl pH 7.5, 150 mM NaCl, 1 mM TCEP) and reactions were started upon addition of PLpro in a final volume of 100 µL and measured at 25 °C. The putative inhibitor compounds were incubated with PLpro enzyme at 10 °C for 6 h. Inhibition kinetics were measured in triplicates over 60 min with one read per minute in two independent experiments. Measured fluorescence values were blank corrected with buffer containing either the ISG15-Rhodamine or the Ub-Rhodamine substrates, respectively.

IC_50_ determination was performed with ISG15-Rhodamine as the substrate at a concentration of 250 nM. The assays were performed as described above, however a gradient concentration of all three natural compounds and GRL-0617 were used in the concentration ranging from 2 to 50 µM in reaction mixture prior to incubation. The IC_50_ values were calculated by the dose-response-inhibition function after the normalization of the enzymatic activity values. Microsoft Excel and GraphPad Prism (version 8.3.1) were used for analyzing the results and preparation of corresponding figures.

### Cytotoxicity assays

Vero cell lines (ATCC® CCL-81™) were cultivated in Dulbecco’s modified Eagle’s medium (DMEM) supplemented with 10% fetal bovine serum (FBS). The cells were seeded in 96-well plates at a density of 3.5 × 10^4^ cells/well, following 24 h incubation at 37 °C and 5% CO_2_ atmosphere. The cell culture media was changed and tenfold serial dilutions of the compounds were added. Cell viability following 72 h treatment of cells with the respective compounds was determined via CellTiter-Glo® Luminescent Cell Viability Assay (Promega), following the manufacturer’s instructions. Luminescent signal was recorded using a CLARIOstar multi-mode microplate reader (BMG Labtech, Germany). Data were obtained from three independent replicates in three biological experiments. Samples deemed to be technical failures and extreme outlier were removed. Wells containing only culture medium served as a control to determine the assay background.

### Antiviral activity assay

Vero cell lines (ATCC® CCL-81™) cultivated in DMEM supplemented with 10% FBS was seeded in 96-well plates at a density of 3.5 × 10^4^ cells/well, following 24 h incubation at 37 °C and 5% CO_2_ atmosphere. The cell culture media was changed and tenfold serial dilution of the compounds were added to the cells. The assays were performed as published previously^4^. Briefly, after 1 h incubation, SARS-CoV-2 strain^69^, diluted in DMEM with 2.5% FBS, was added to the cells at a MOI of 0.01 and allowed absorption for 1 h. The viral inoculum was removed, and cells were gently washed with phosphate-buffered saline (PBS) without calcium and magnesium. Fresh DMEM with 2.5% FBS containing the compounds was added back onto the cells. Cell culture supernatant was harvest 42 h post-infection and viral RNA was purified using MagMAX™ Viral/Pathogen Nucleic Acid Isolation Kit (Thermo Fisher Scientific). The samples were processed using the semi-automated NucliSENS® easyMag® platform (bioMérieux, Lyon, France), following the manufacturer's instructions. All SARS-CoV-2 infections were performed in a biosafety level 3 laboratory at the Institute of Biomedical Sciences, University of São Paulo, Brazil. The viral titers were determined by the qRT-PCR method using AgPath-ID™ One-Step RT-PCR Kit (Thermo Fisher Scientific) and a sequence of primers and probe for the E gene^70^. The viral titers were calculated using a standard curve generated with serial dilutions of a template known concentration and expressed in TCID_50_/mL. Infected cells with the addition of 0.5% DMSO were used as control. IC_50_-values were calculated by fitting the data using GraphPad Prism version 8.00 (GraphPad Software, La Jolla California USA). Data were obtained from four independent replicates in two biological experiments. Samples deemed to be technical failures and extreme outlier were removed.

### Cytopathic effect inhibition

Vero cell lines (ATCC® CCL-81™) cultivated in DMEM supplemented with 10% FBS were seeded in 96-well plates at a density of 3.5 × 10^4^ cells/well, following 24 h incubation at 37 °C and 5% CO_2_ atmosphere. The cell culture media was changed and tenfold serial dilution of the compounds were added to the cells. The cells were infected at MOI 0.01 and the cytopathic effect (CPE) inhibition following 42 h treatment of cells with the respective compounds was determined via CellTiter-Glo® Luminescent Cell Viability Assay (Promega). Luminescent signal was recorded using a CLARIOstar multi-mode microplate reader (BMG Labtech, Germany). Data were obtained from three independent replicates in one biological experiment. Samples deemed to be technical failures and extreme outlier were removed.

The luminescent-based assay measures the inhibition of SARS-CoV2–induced cytopathic effect (CPE) in Vero cell line (ATCC® CCL-81™)^49^. Percent cytopathic effect (CPE) inhibition was defined as [(test compound−virus control)/(cell control−virus control)] × 100. IC_50_ values were fitted by sigmoidal function using GraphPad Prism version 8.00 (GraphPad Software, La Jolla California USA).

### Nano differential scanning fluorimetry

nDSF measurements were performed applying a Nanotemper Prometheus NT.48 fluorimeter (Nanotemper) operated by PR.ThermControl software and using Prometheus Premium grade capillaries (Nanotemper). The excitation power was adjusted to obtain fluorescence signals above 2000 RFU for a wavelength range of 330 and 350 nm. For all measurements a PLpro concentration of 50 μM in the buffer consisting of 20 mM Tris-HCl, 150 mM NaCl, 0.5 mM TCEP, pH 8.0 was used, and varying ligand concentrations. For the ligand HE9 0.5% DMSO was added to ensure solubility. For the fluorescence titrations 1:1 dilution series with 16 points of ligands was designed and after the corresponding protein solutions were added. Ligand concentrations range from 20 mM to 610 nM for HBA and 5 mM to 153 nM for HE9. After incubation of 30 min, the solutions were transferred to capillaries and utilized for the measurements. Data were analysed and visualized applying self-written python scripts using the Python modules Numpy, Matplotlib, Scipy, and Pandas and a publicly available SPC data analysis platform.^46^ The fluorescence values *F* vs. the ligand concentration *[L]*_*0*_ of HBA and HE9 were fitted with a simple 1:1 binding model using the Eqs. (1) and (2) below:

1 $$F({[L]}_{0})={F}_{{{{{\rm{upper}}}}}}+({F}_{{{{{\rm{upper}}}}}}-{F}_{{{{{\rm{lower}}}}}})* (1-a({[L]}_{0}))$$

2 $$a({[L]}_{0})=\left({[P]}_{0}-{K}_{d}-{[L]}_{0}+ \sqrt{{({[P]}_{0}+{[L]}_{0}+{K}_{d})}^{2}-4 * {[P]}_{0} * {[L]}_{0}}\right)/(2* {[P]}_{0})$$

### SARS-CoV-2 Mpro inhibition assays and IC_50_ determination

Activity assays were performed for SARS-CoV-2 Mpro^16,71^ utilizing the three natural compounds, aiming to characterize the specificity of the compounds towards PLpro. The assays were performed applying a total reaction volume of 50 µL using non-binding, black bottom, 96-well plates and the relative fluorescence was measured utilizing a Tecan Infinite M plus plate reader (Tecan Group Ltd, Switzerland) using optical settings specific for the substrate 2-AbzSAVLQSGTyr(3-NO2)R-OH (Biotrend). The corresponding fluorophore has an excitation and emission at 355 nm and 460 nm wavelength respectively. The substrate and Mpro were used at a final concentration of 5 µM and 75 nM respectively. The known Mpro inhibitor (GC-376) was used as positive control throughout the assay. SARS-CoV-2 Mpro and substrates were diluted in assay buffer (20 mM Tris-HCl pH 7.3, 150 mM NaCl, 1 mM EDTA, 1 mM DTT) and reactions were initiated upon addition of Mpro in a final volume of 50 µL and were measured at 25 °C. The three compounds were incubated prior to the experiments with Mpro at 10 °C for 6 h, as accomplished also for the PLpro activity assays. Fluorescence values were measured for 15 min with one read out per minute.

IC_50_ determination was performed applying the same substrate at a concentration of 5 µM and the corresponding assays were performed as described above. A concentration gradient in a range of 1 nM–150 µM was used for all three natural compounds and GC-376. The IC_50_ values were calculated applying a dose-response-inhibition function after normalization of the enzymatic activity values. Microsoft Excel and the software Origin (OriginLab) were used for analyzing the data obtained to prepare the corresponding figures.

### Reporting summary

Further information on research design is available in the Nature Research Reporting Summary linked to this article.

## Supplementary information

Supplementary Materials

Reporting Summary

## Data Availability

Coordinates and structure factors were deposited in the Protein Data Bank PDB, with codes: 7NFV (PLpro), 7OFS (PLpro in complex with YRL, 4-(2-hydroxyethyl)phenol), 7OFT (PLpro in complex with HBA, p-hydroxybenzaldehyde) and 7OFU (PLpro in complex with HE9, 3, 4-dihydroxybenzoic acid, methyl ester).
