# Supplementary material for: Antiviral activity of natural phenolic compounds in complex at an allosteric site of SARS-CoV-2 papain-like protease
Source: Commun Biol. 2022 Aug 11;5:805. doi: 10.1038/s42003-022-03737-7 (PMC9366811; doi:10.1038/s42003-022-03737-7)
Supplement: Supplementary file 1 — Supplementary Materials [file 42003_2022_3737_MOESM1_ESM.pdf]

Supplementary Materials for

Antiviral activity of natural phenolic compounds in complex at an allosteric site of SARS-CoV-2 papain-like protease

Vasundara Srinivasan(1,*), Hévila Brognaro(1), Prince R. Prabhu(1,2), Edmarcia Elisa de Souza(3), Sebastian Günther(4), Patrick Y. A. Reinke(4), Thomas J. Lane(2,4), Helen Ginn(5), Huijong Han(6), Wiebke Ewert(4), Janina Sprenger(4), Faisal H. M. Koua(4), Sven Falke(1,4), Nadine Werner(1), Hina Andaleeb(1,7), Najeeb Ullah(1,7), Bruno Alves Franca(1), Mengying Wang(1), Angélica Luana C Barra(1,8), Markus Perbandt(1), Martin Schwinzer(1), Christina Schmidt(6), Lea Brings(6), Kristina Lorenzen(6), Robin Schubert(6), Rafael Rahal Guaragna Machado(9), Erika Donizette Candido(9), Danielle Bruna Leal Oliveira(9,10), Edison Luiz Durigon(9,11), Stephan Niebling(12), Angelica Struve Garcia(12), Oleksandr Yefanov(4), Julia Lieske(4), Luca Gelisio(4), Martin Domaracky(4), Philipp Middendorf(4), Michael Groessler(4), Fabian Trost(4), Marina Galchenkova(4), Aida Rahmani Mashhour(4), Sofiane Saouane(13), Johanna Hakanpää(13), Markus Wolf(14), Maria Garcia Alai(12), Dusan Turk(15,16), Arwen R. Pearson(2,17), Henry N. Chapman(2,4,18), Winfried Hinrichs(19), Carsten Wrenger(3), Alke Meents(4), Christian Betzel(1,2*).

1) Universität Hamburg, Department of Chemistry, Institute of Biochemistry and Molecular Biology, Laboratory for Structural Biology of Infection and Inflammation, Build. 22a, c/o DESY, 22607 Hamburg, Germany.

2) Hamburg Centre for Ultrafast Imaging (CUI), Universität Hamburg, Luruper Chaussee 149, 22761, Hamburg, Germany.

3) Department of Parasitology, Institute of Biomedical Sciences at the University of São Paulo, São Paulo, Brazil.

4) Center for Free-Electron Laser Science, CFEL, Deutsches Elektronen Synchrotron DESY, Notkestrasse 85, 22607 Hamburg, Germany.

5) Diamond Light Source Ltd. Diamond House, Harwell Science and Innovation Campus, Didcot, OX11 0DE, UK.

6) European XFEL GmbH. Holzkoppel 4, 22869 Schenefeld, Germany.

7) Department of Biochemistry, Bahauddin Zakariya University Multan-60800, Punjab, Pakistan.

8) Pólo TerRa, São Carlos Institute of Physics, University of São Paulo, São Carlos, Brazil.

9) Department of Microbiology, Institute of Biomedical Sciences at the University of São Paulo, São Paulo, Brazil.

10) Clinical Laboratory, Hospital Israelita Albert Einstein, São Paulo, Brazil.

11) Scientific Platform Pasteur USP, São Paulo, Brazil.

12) European Molecular Biology Laboratory Hamburg, c/o DESY, Notkestrasse 85, 22607 Hamburg. Germany.

13) Photon Science, Deutsches Elektronen Synchrotron (DESY), Notkestrasse 85, 22607, Hamburg, Germany.

14) Fraunhofer Institute for Translational Medicine and Pharmacology (ITMP), Schnackenburgallee114, 22525 Hamburg, Germany.

15) Department of Biochemistry & Molecular & Structural Biology, Jozef Stefan Institute, Jamova 39,1 000 Ljubljana, Slovenia.

16) Centre of excellence for Integrated Approaches in Chemistry and Biology of Proteins (CIPKEBIP), Jamova 39, 1 000 Ljubljana,Slovenia.

17) Universität Hamburg, Institut für Nanostruktur- und Festkörperphysik, Luruper Chaussee 149, 22761, Hamburg, Germany.

18) Department of Physics, Universität Hamburg, Luruper Chaussee 149, 22761, Hamburg, Germany.

19) Universität Greifswald, Institute of Biochemistry, Felix-Hausdorff-Str. 4, 17489 Greifswald, Germany.

Correspondence to: vasundara.srinivasan@chemie.uni-hamburg.de, christian.betzel@uni-hamburg.de

This PDF file includes:

Supplementary Notes
Figures. S1 to S16

Supplementary Note 1

Extraction, Isolation and Purification of p-hydroxy-benzaldehyde (HBA)

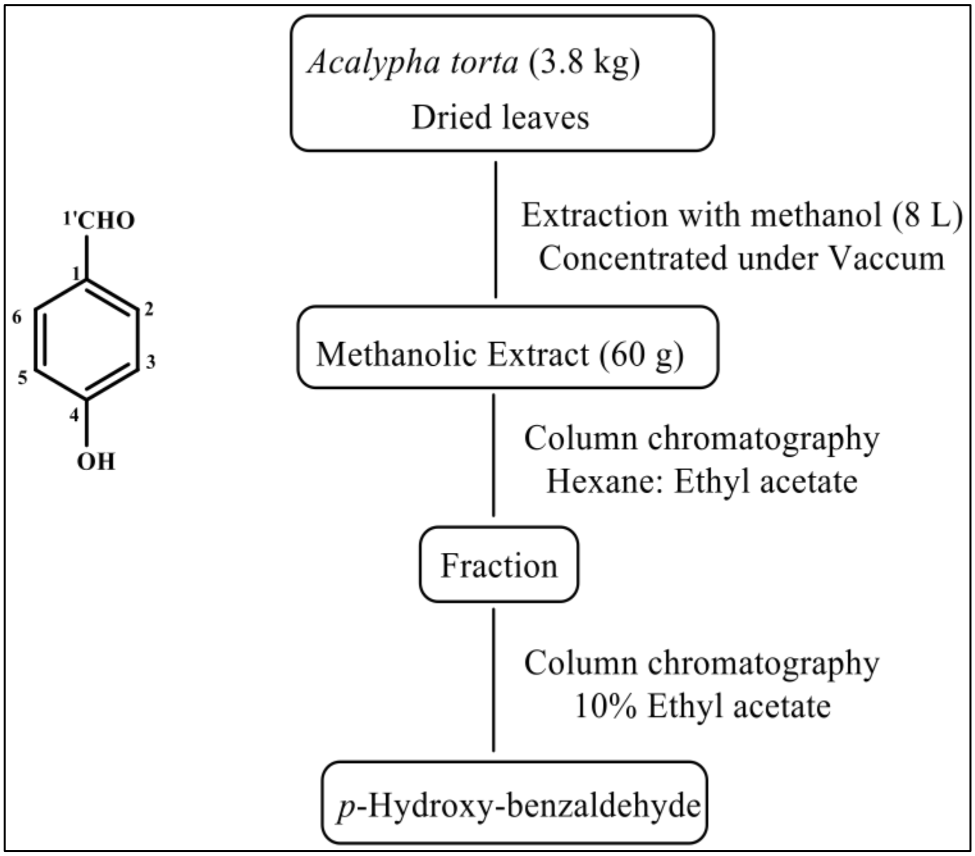

Leaves of *Acalypha torta* were collected from the flower Kingdom in Ibada, Oyo State, Nigeria. The compound p-hydroxy-benzaldehyde (HBA) was extracted, isolated and purified following the scheme above. The compound was analyzed by Electron Ionization Mass Spectrometry (EI-MS) that showed a peak with m/z 122, with a fragmentation pattern at m/z 121, 97, 95, 81, 69, and 55. The NMR spectrum is shown below. The MS and NMR spectra of HBA corresponds to the data published before[1].

Figure S1. NMR spectra of p-Hydroxy-benzaldehyde (HBA)

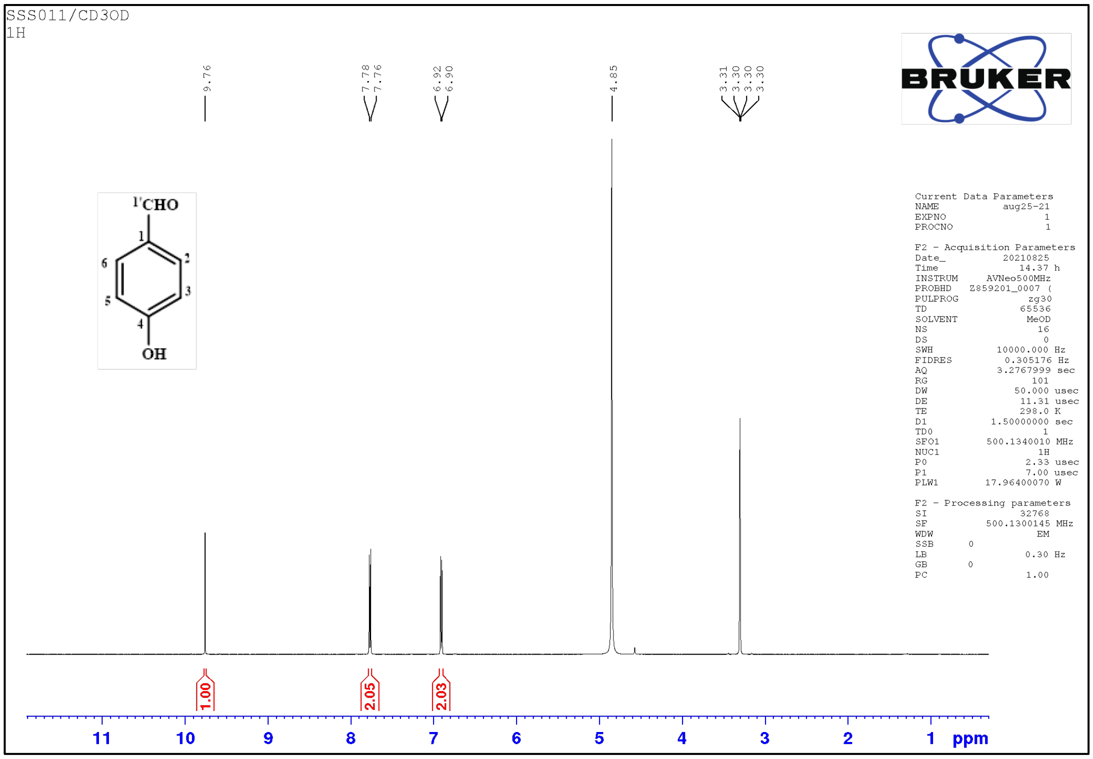

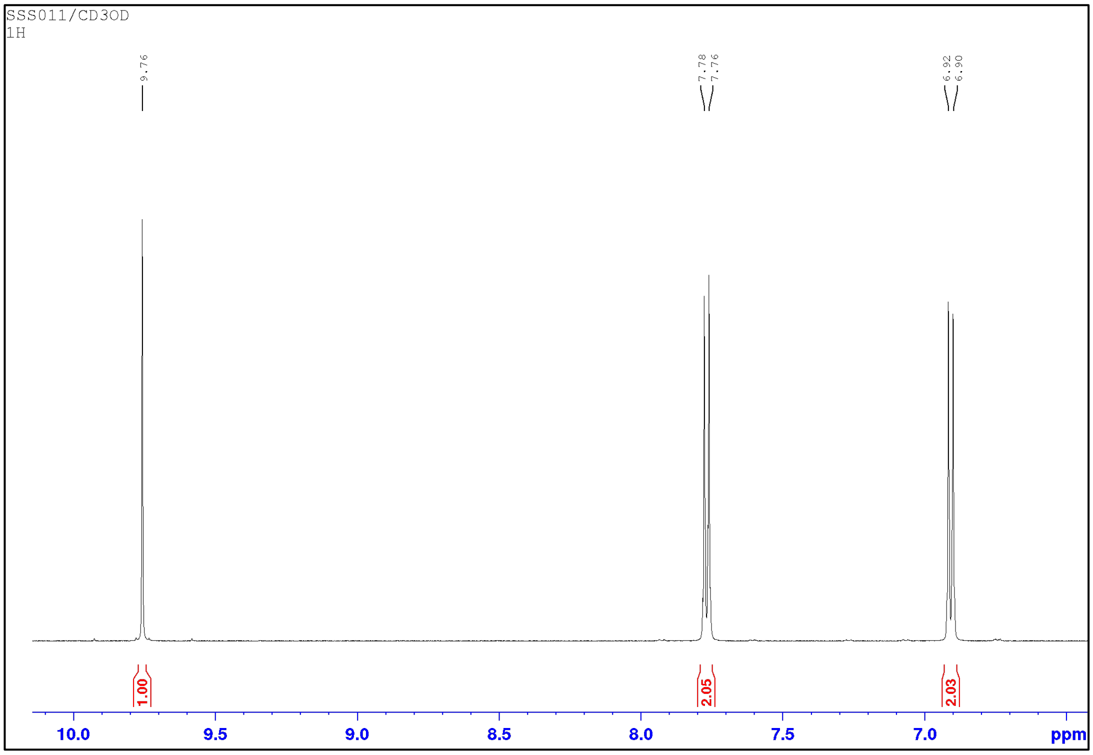

Supplementary Note 2

Extraction, Isolation and Purification of 4-(2-Hydroxyethyl) Phenol (YRL)

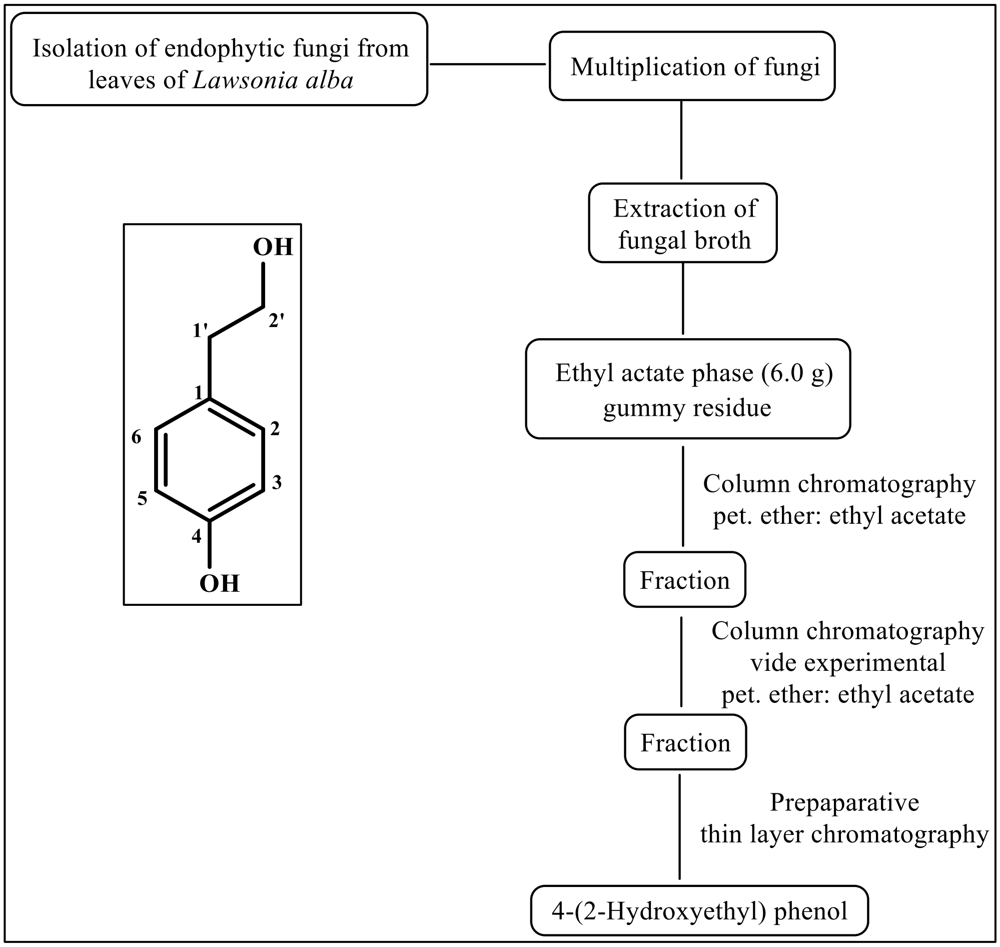

Endophytic fungi from leaves of *Lawsonia alba* were collected at ICCBS, University of Karachi, Karachi, Pakistan. Extraction, isolation and purification of 4-(2-hydroxyethyl) phenol (YRL) is shown in the scheme above. The compound YRL was analyzed by mass spectrometry that showed a peak at m/z corresponding to a mass of 138.07 Da. The NMR spectrum is shown below. Spectral data obtained for the compound were identical to previously reported data[2].

Figure S2. NMR spectra of 4-(2-Hydroxyethyl) Phenol (YRL)

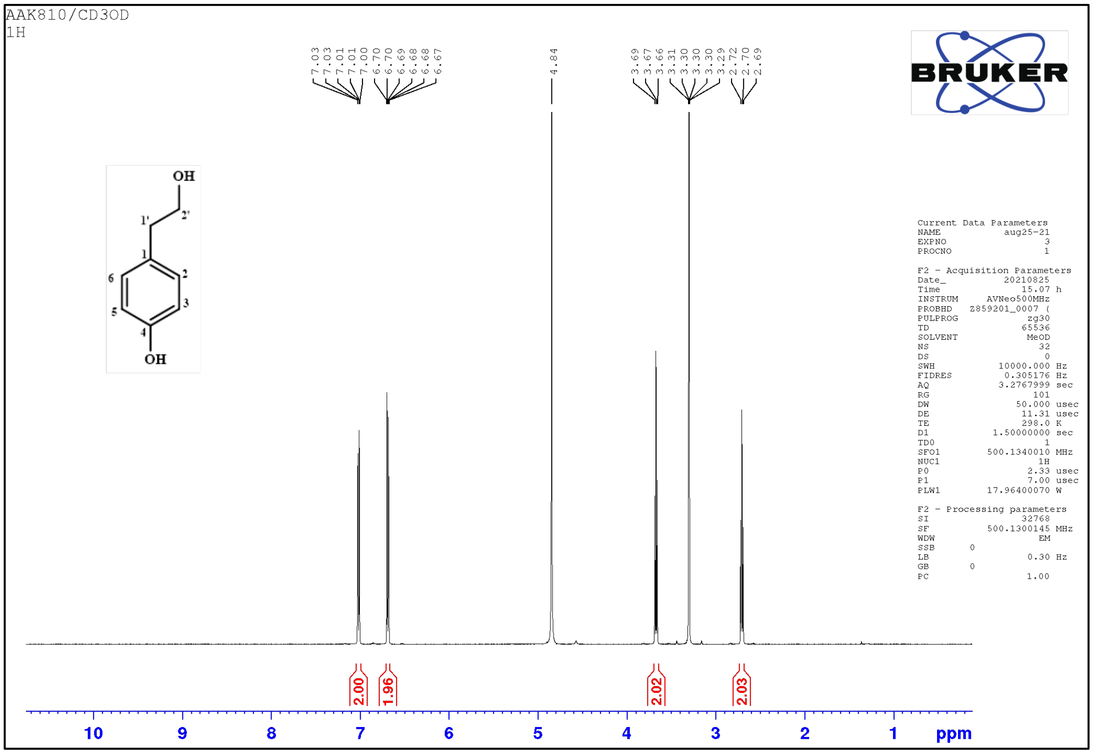

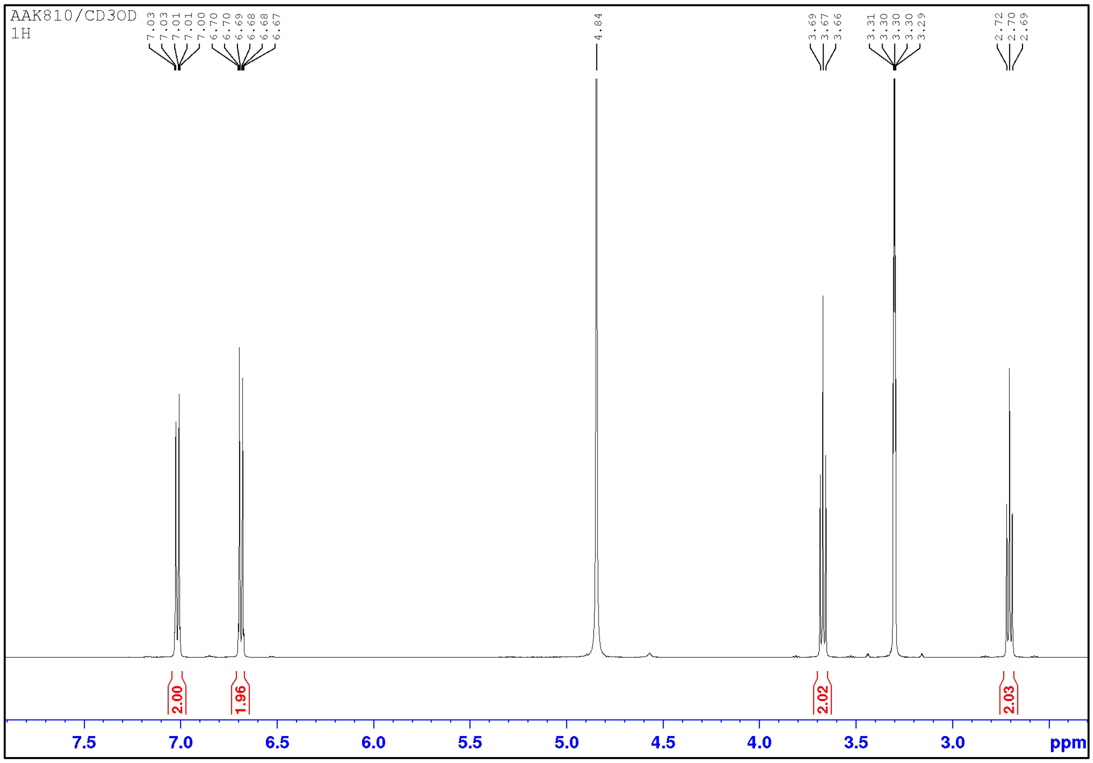

Supplementary Note 3

Extraction, Isolation and Purification of Methyl-3,4-dihydroxybenzoate (HE9)

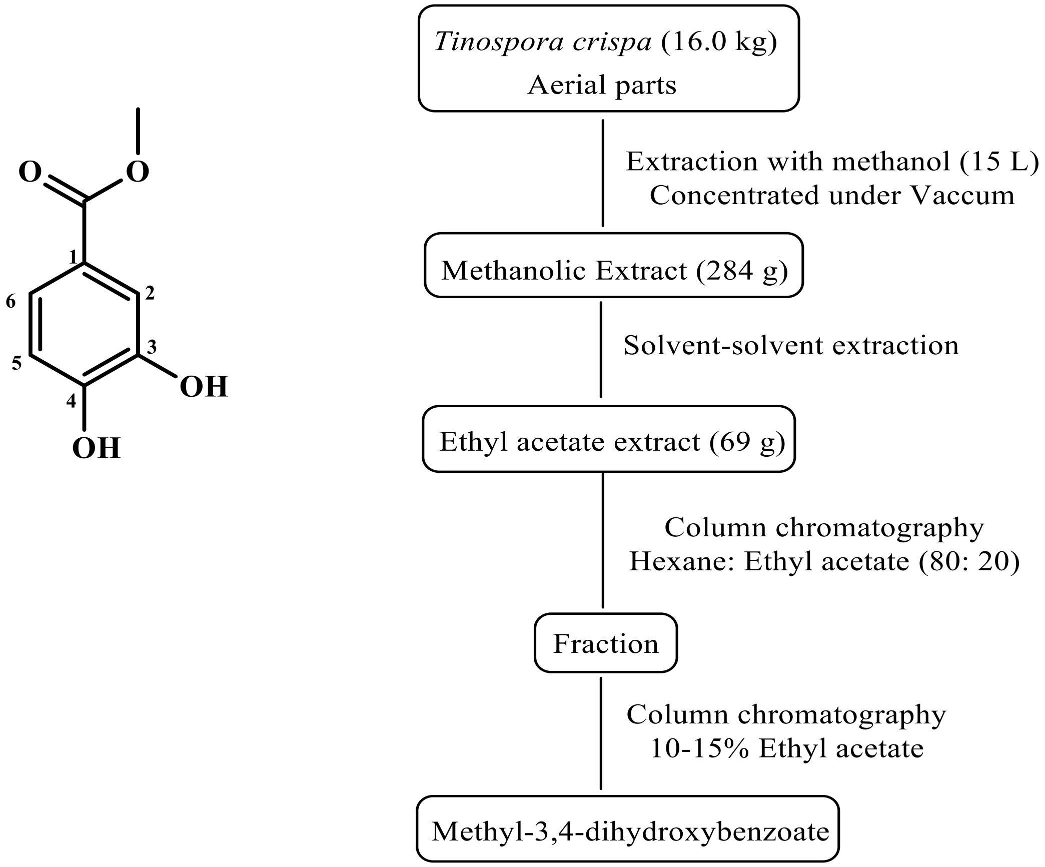

Aerial parts of *Tinospora crispa Meirs* were collected from the herbal gardens of the Laboratory of Natural Products, University of Putra, Malaysia. Extraction, isolation and purification of methyl-3,4-dihydroxybenzoate (HE9) is shown in the scheme above. The compound HE9 was analyzed by mass spectrometry that showed a peak at m/z corresponding to 168.04 Da. The NMR spectrum is shown below. The spectra are in agreement to previously reported data of the same compound isolated from *Schisandra verruculosa*[3].

Figure S3. NMR spectra of Methyl-3,4-dihydroxybenzoate (HE9)

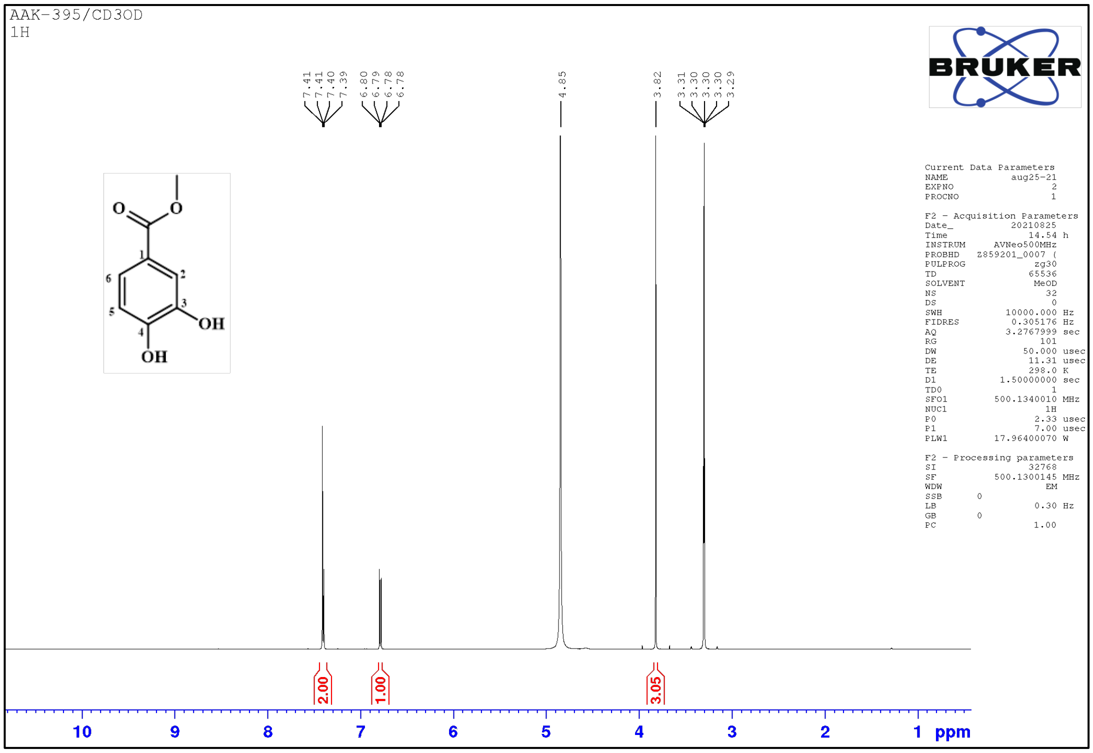

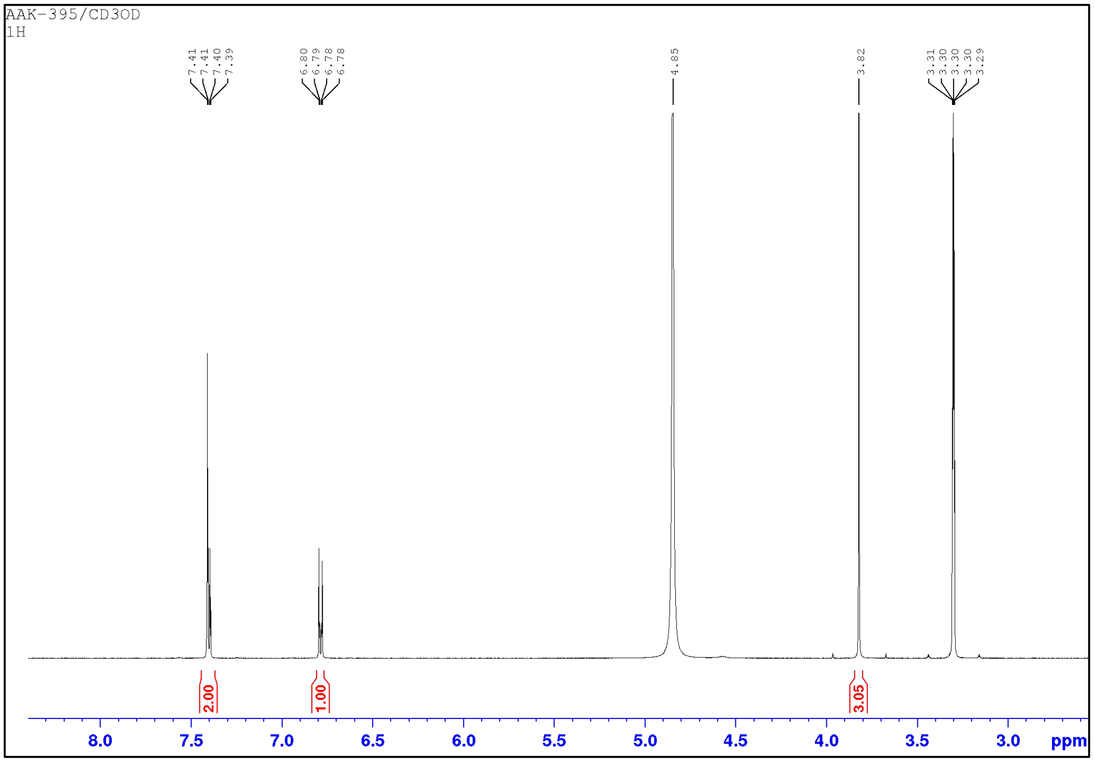

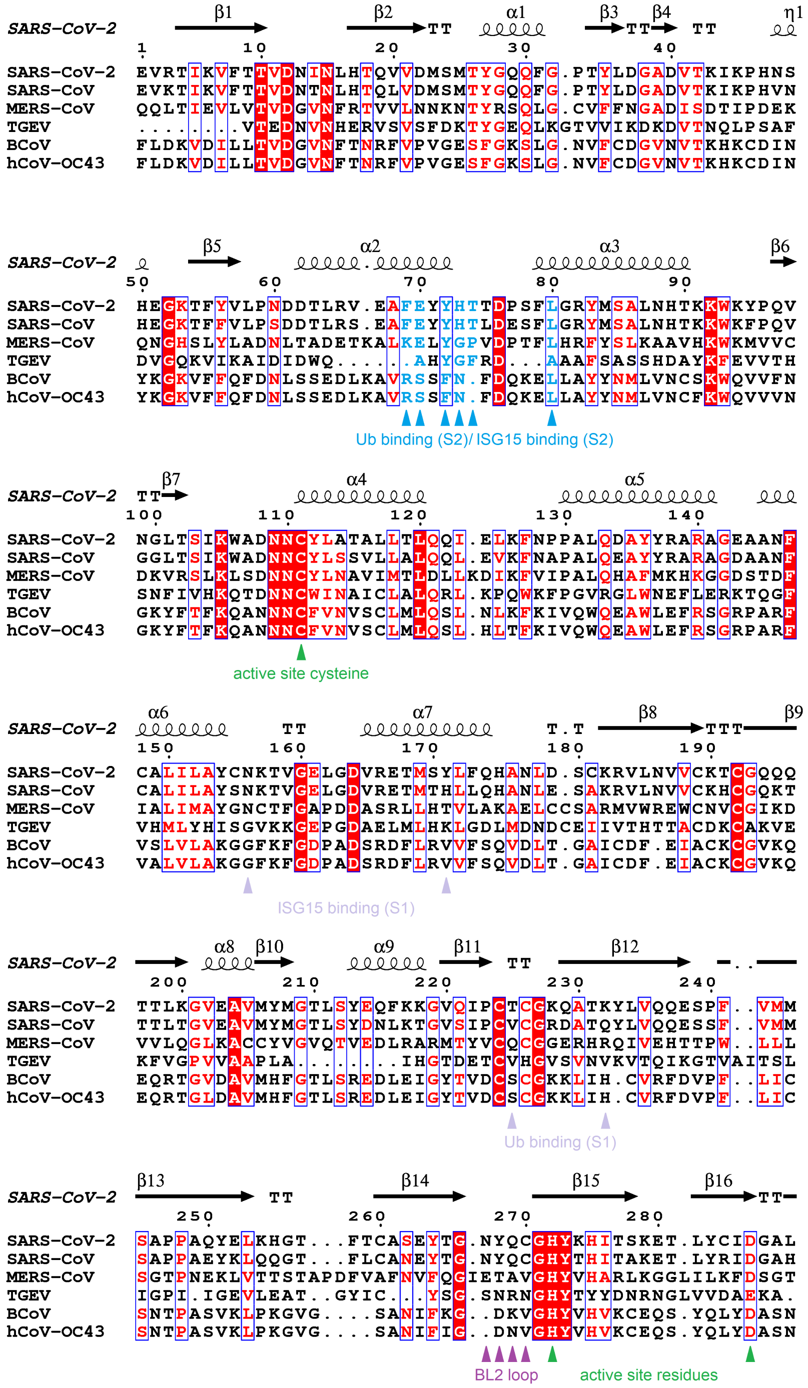

Figure S4.

Sequence alignment of PLpro domains from selected α-, β- and related γ- coronaviruses

Sequences of PLpro from SARS-CoV-2, ID: P0DTC1; SARS-CoV, ID: P0C6X7; MERS-CoV (Middle East Respiratory Syndrome Coronavirus), ID: K9N638; TGEV (Transmissible Gastroenteritis Virus), ID: P0C6V2; BCoV (Bovine Coronavirus), ID: P0C6W7; hCoV-OC43 (Human Coronavirus OC43), ID: P0C6X6 are aligned using Espript 3[4]. The secondary structure and sequence numbering based on our crystal structure of PLpro (PDB code 7NFV) is depicted on the top of the alignment. Active site residues are indicated in green triangles, the blocking

loop in purple, the ISG15 binding S1 site in dark red and the ISG15 S2 allosteric binding site overlapping with the inhibitor binding site is shown in light blue.

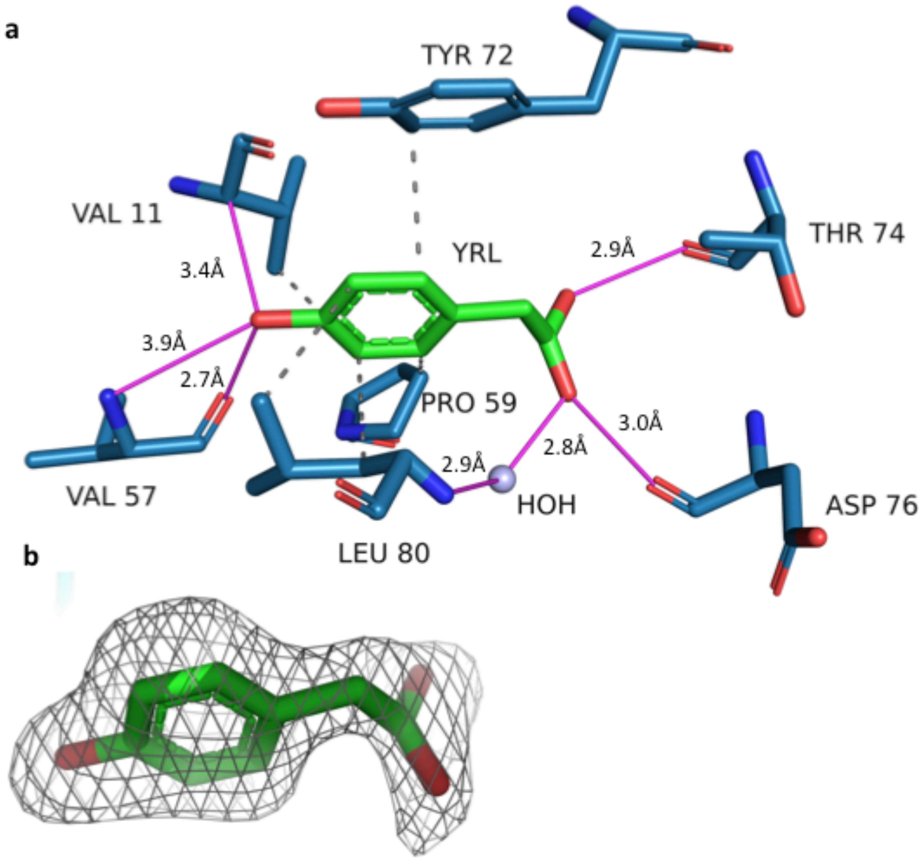

Figure S5.

Interaction network of the three compounds in PLpro complex structures

a. YRL

a. YRL interactions with PLpro are mediated by hydrogen bonds of the phenolic hydroxyl to amino acid residues Val 11 and Val 57. The aliphatic hydroxyl substituent of YRL has an alternate conformation with equal occupancy in the complex. Both conformations are stabilized by hydrogen bonds to backbone carbonyl oxygens of Thr 74 and Asp 76. Hydrophobic interactions (black dashed line) with Val 11, Pro 59, Tyr 72 and Leu 80 complete the interaction network. YRL is represented as green sticks and the amino acid residues in blue sticks.

b. Fo-Fc electron density map contoured at a sigma level 3.0 for YRL

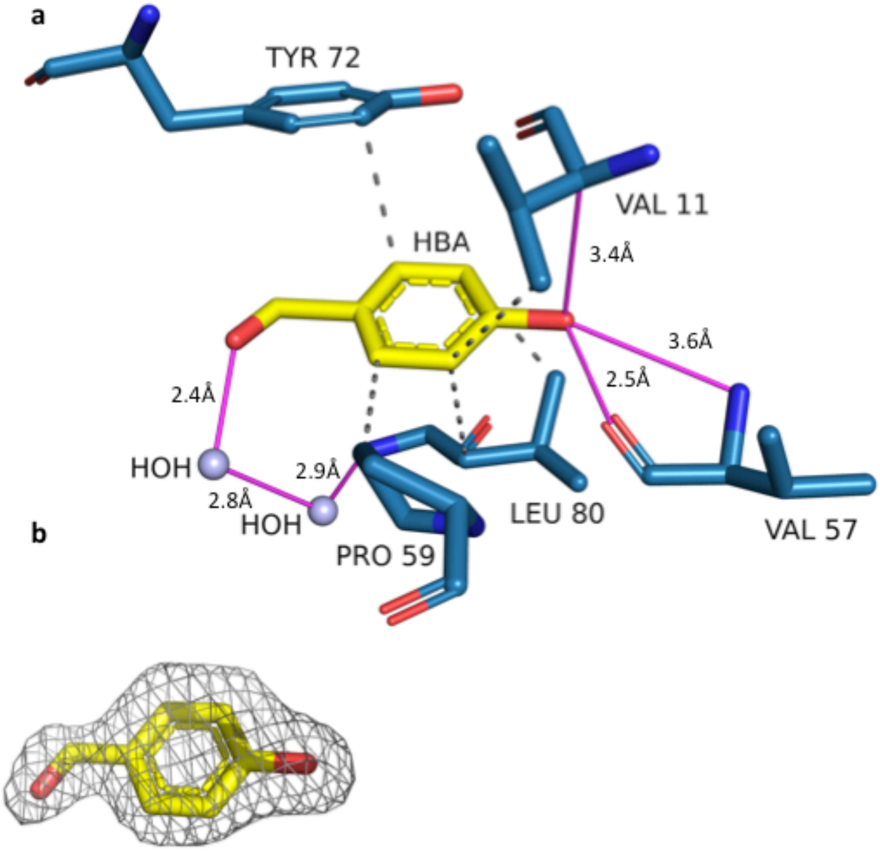

Figure S6.

Interaction network of the three compounds in PLpro complex structures

a. HBA binds in a hydrophobic cavity with interactions to Val 11, Pro 59, Tyr 72 and Leu 80. The binding pattern is similar to that of YRL, including the hydrogen bond to Val 57. HBA is represented as yellow sticks and the amino acid residues in blue sticks.

b. Fo-Fc electron density map contoured at a sigma level 3.0 for HBA.

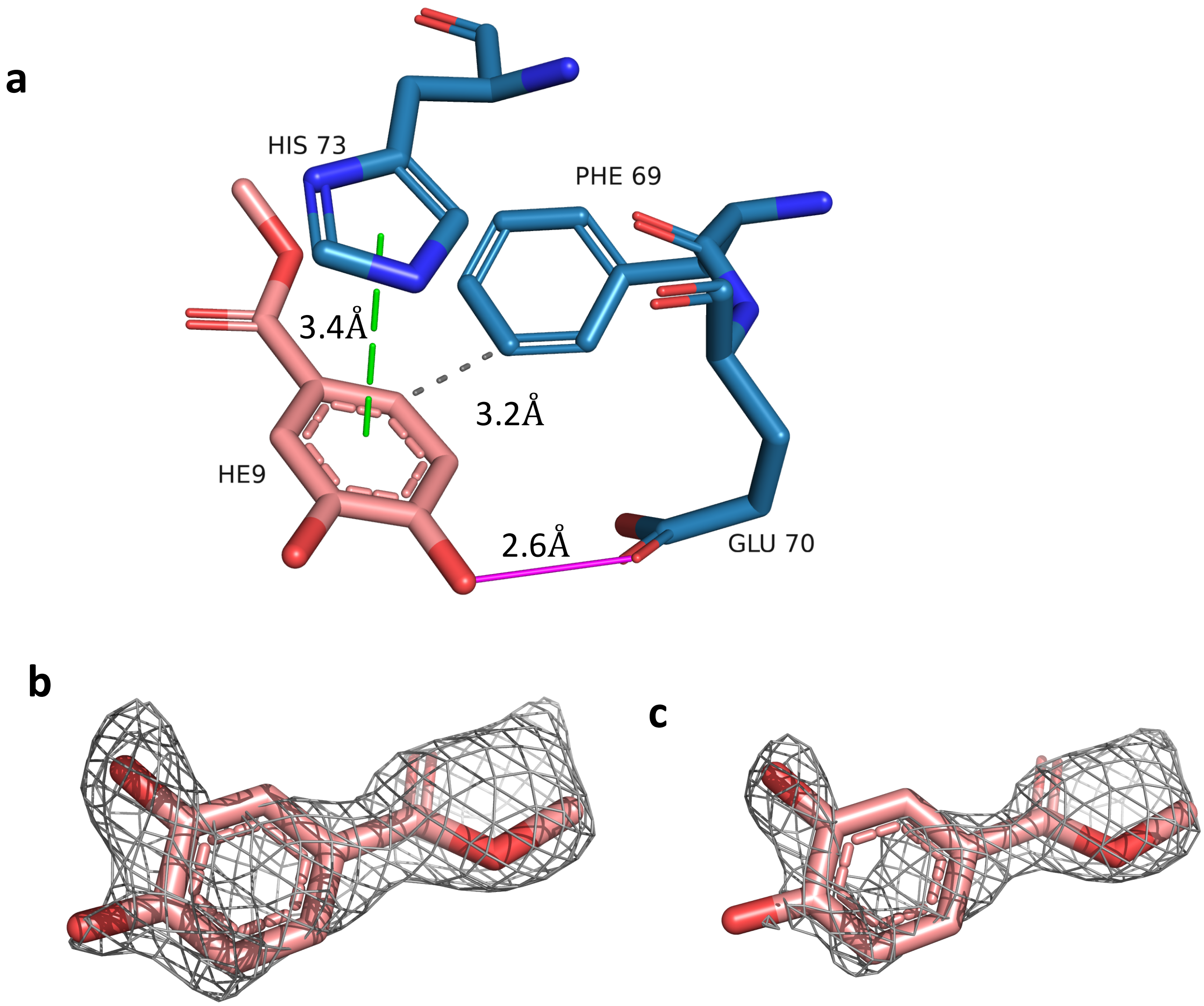

Figure S7.

Interaction network of the three compounds in PLpro complex structures

a. HE9 binding to PLpro is mediated by hydrophobic interaction with Phe 69 and π-stacking (green dashed line) with His 73. A hydrogen bond with the side chain of Glu 70 completes the interaction network of HE9 to PLpro. HE9 is represented as pink sticks and the amino acid residues in blue sticks.

b. Fo-Fc electron density map contoured at a sigma level 2.5 for HE9.

c. Fo-Fc electron density map contoured at a sigma level 3.0 for HE9.

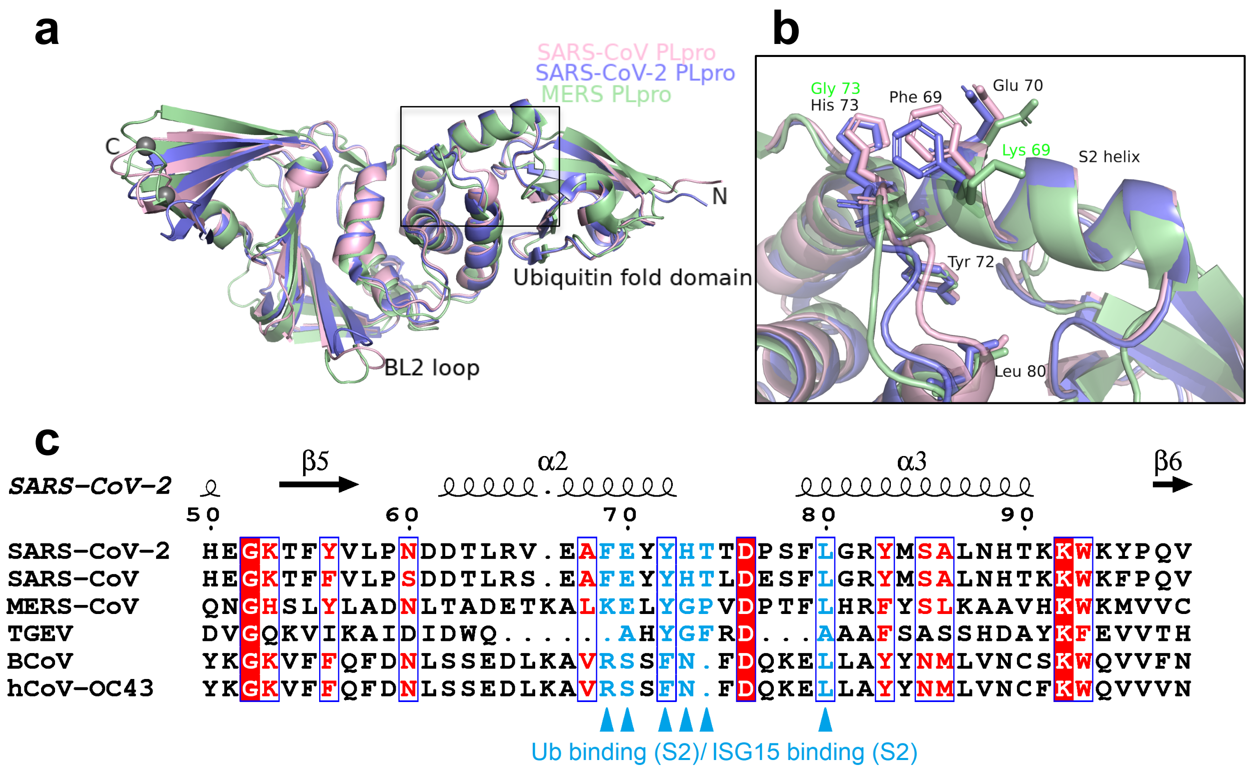

Figure S8.

Comparison of the ISG15-S2 allosteric binding site in SARS-CoV, SARS-CoV-2 and MERS-CoV PLpro

a. Superposition of the crystal structures of SARS-CoV PLpro (PDB: 2FE8 in light pink), SARS-CoV-2 PLpro (PDB: 7NFV in slate blue) and MERS-CoV PLpro (PDB: 4RNA in light green). The zinc fingers domain, ubiquitin-fold domain and the blocking loop 2 (BL2) are the most variable regions among the three PLpro structures.

b. The S2 binding ISG15 site in PLpro consisting of residues Phe 69, Glu 70, Tyr 72, His 73 and Leu 80 is conserved between SARS-CoV and SARS-CoV-2.

c. The sequence alignment for the region corresponding to the S2 ISG15 binding site shows that the MERS-CoV differs in critical binding residues; Phe 69 in SARS-CoV-2 PLpro is replaced by a lysine residue (F69K), Tyr 72 to a leucine residue (Y72L) and His 73 to a glycine residue (H73G).

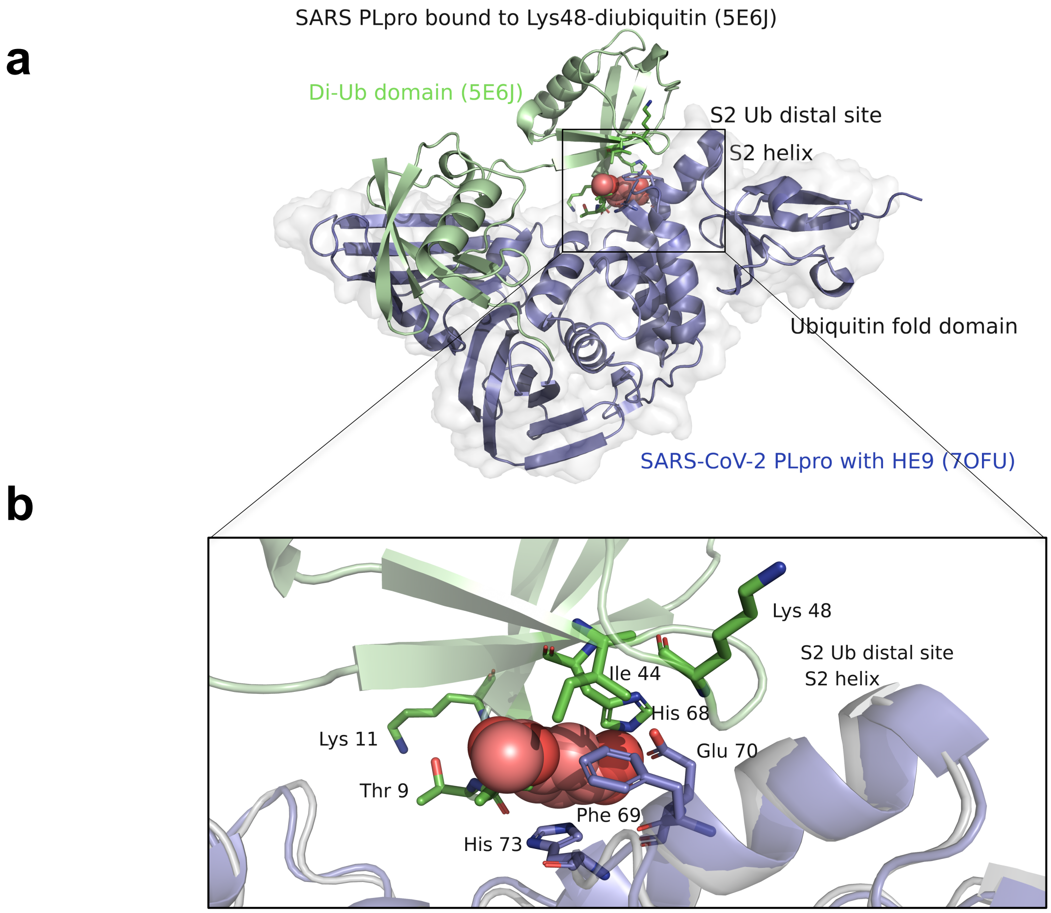

Figure S9.
The binding of the three natural compounds disrupts interaction of the K48-linked di-ubiquitin molecule to PLpro

a. Superposition of the crystal structures of SARS-CoV PLpro complex with Lys48 linked di-Ub (PDB code 5E6J, PLpro in grey, di-Ubiquitin domain in green) with SARS-CoV-2 PLpro+HE9 (PDB code 7OFU, in slate blue). The natural compound (HE9) is represented with red spheres.

b. Close-up view of the S2-Ub binding site. The di-ubiquitin molecule is shown in green cartoon representation with the interacting residues Lys 11, Ile 44 and Lys 48 shown in sticks. The bound inhibitor compound HE9 (red spheres) clearly prevents the binding of the di-ubiquitin molecule in the S2 binding site of PLpro.

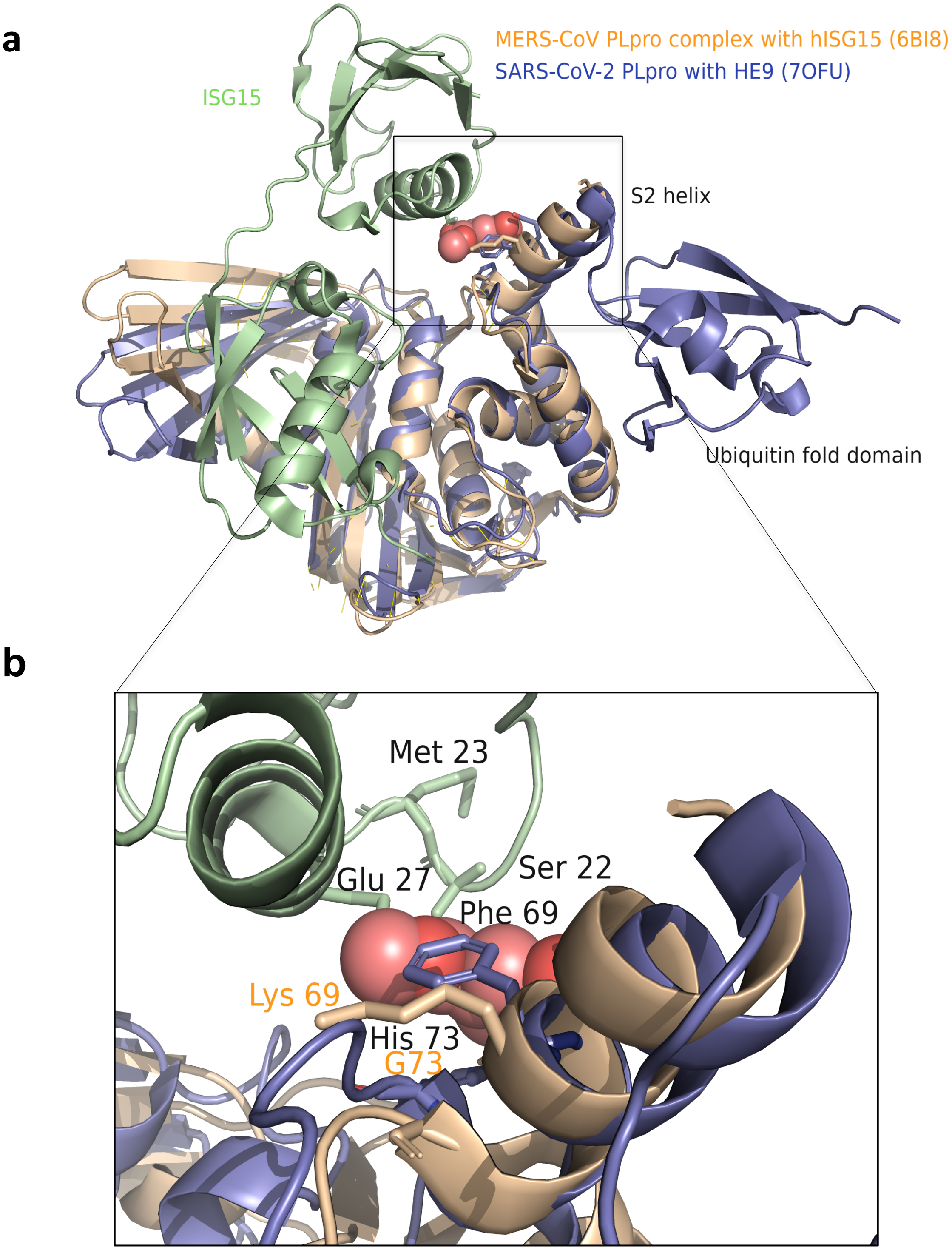

Figure S10.
The binding of HE9 prevents interaction of the ISG15 molecule in MERS-Co-V PLpro

a. Superposition of the crystal structures of MERS-CoV PLpro complex with ISG15 (PDB code 6BI8, PLpro in wheat, ISG15 molecule in green) with SARS-Cov-2 PLpro+HE9 (PDB code 7OFU, in slate blue). The natural compound (HE9) is represented as spheres.

b. Close-up view of the S2-Ub binding site. The ISG15 molecule is shown in green cartoon representation with the interacting residues Ser 22, Met 23 and Glu 27 shown in sticks. The bound inhibitor compound HE9 (red spheres) clearly blocks the binding of the ISG15 molecule to the S2 binding site of MERS-CoV PLpro. The Phe 69 to Lys and His 73 to Gly change contribute to the regional different surface charge properties of MERS-CoV PLpro.

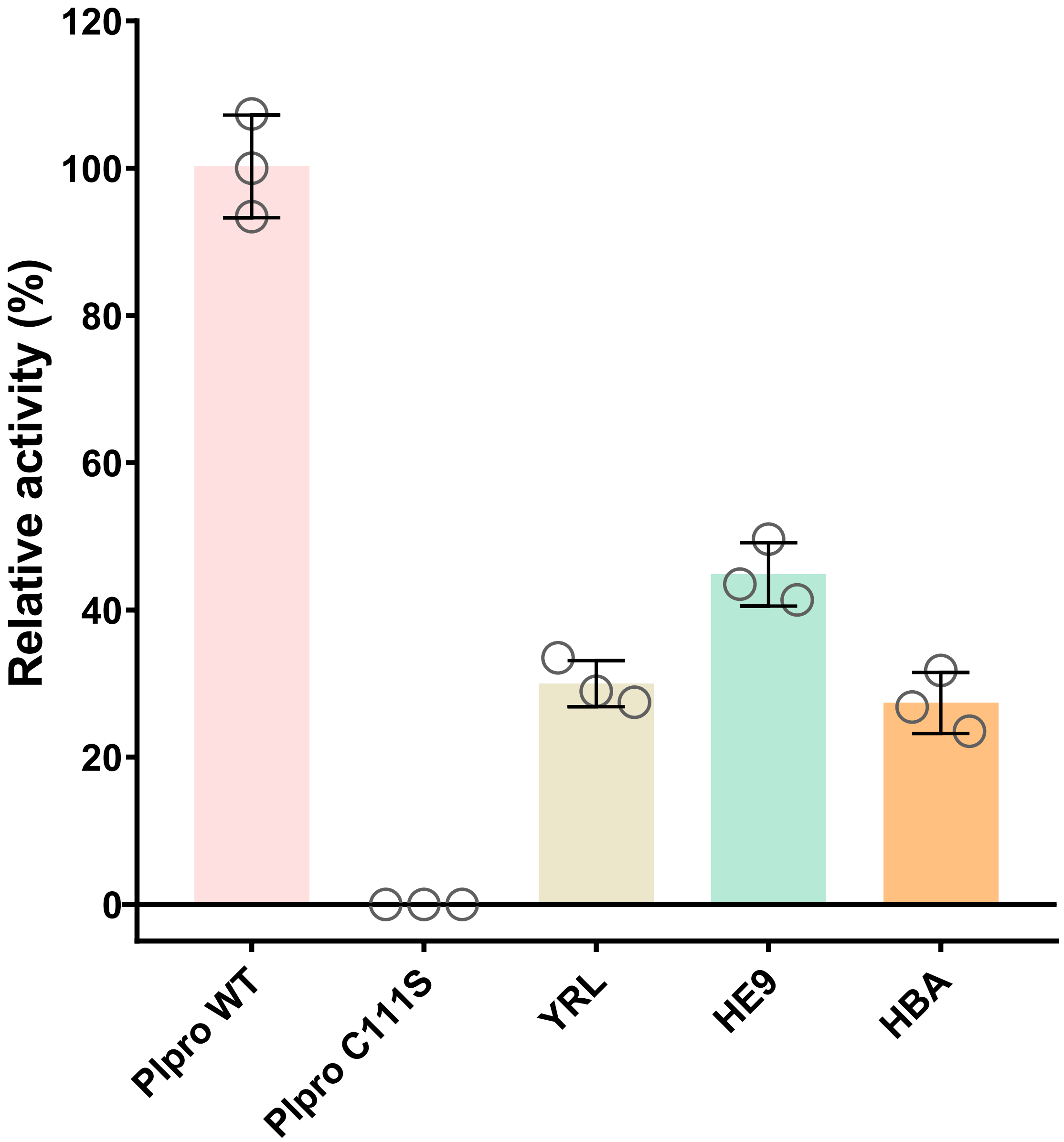

Figure S11.
Inhibition of PLpro by the three natural compounds in deISGylation assay with ISG15-Rh substrate and comparison with catalytically inactive PLpro mutant (C111S)

Wild type PLpro (WT PLpro) at 10nM concentration represents 100% of deISGylation activity and the three natural compounds YRL, HBA and HE9 at 50 µM show clear inhibition of PLpro enzymatic activity. As experimental control, catalytically inactive PLpro mutant C111S at 10nM concentration was used. ISG15-Rh at a concentration of 100nM was used as the substrate. Measured fluorescence values were blank corrected with buffer containing substrate and were measured in triplicates over 60 min with one read per minute.

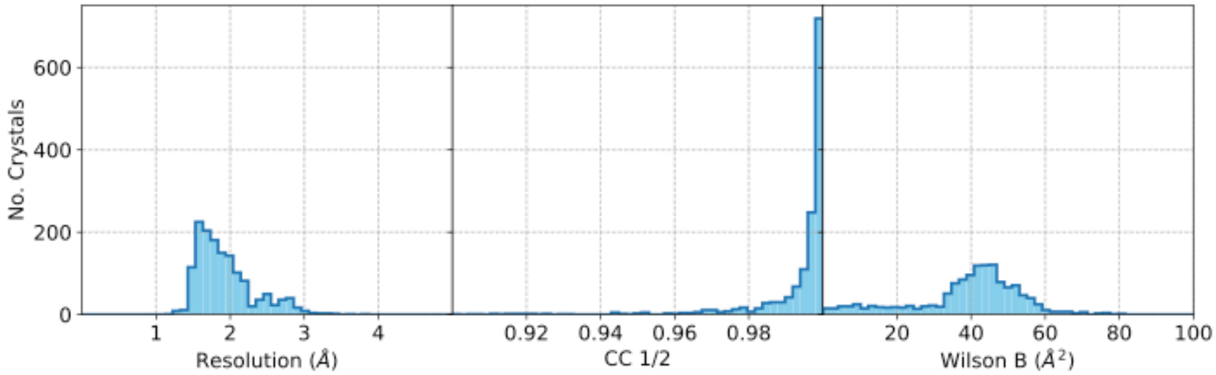

Figure S12:
Data quality indicators of the diffraction datasets by X-ray screening of a library of 500 natural compounds

Approximately 2000 crystals of PLpro co-crystallized with the library of 500 compounds were harvested for data collection. More than one dataset per compound was collected that resulted in about 2500 datasets. A total of 1469 datasets were processed with the previously established automatic pipeline[5]. Data quality indicators, resolution (left), $CC_{1/2}$ (middle) and Wilson B-factor (right) are shown.

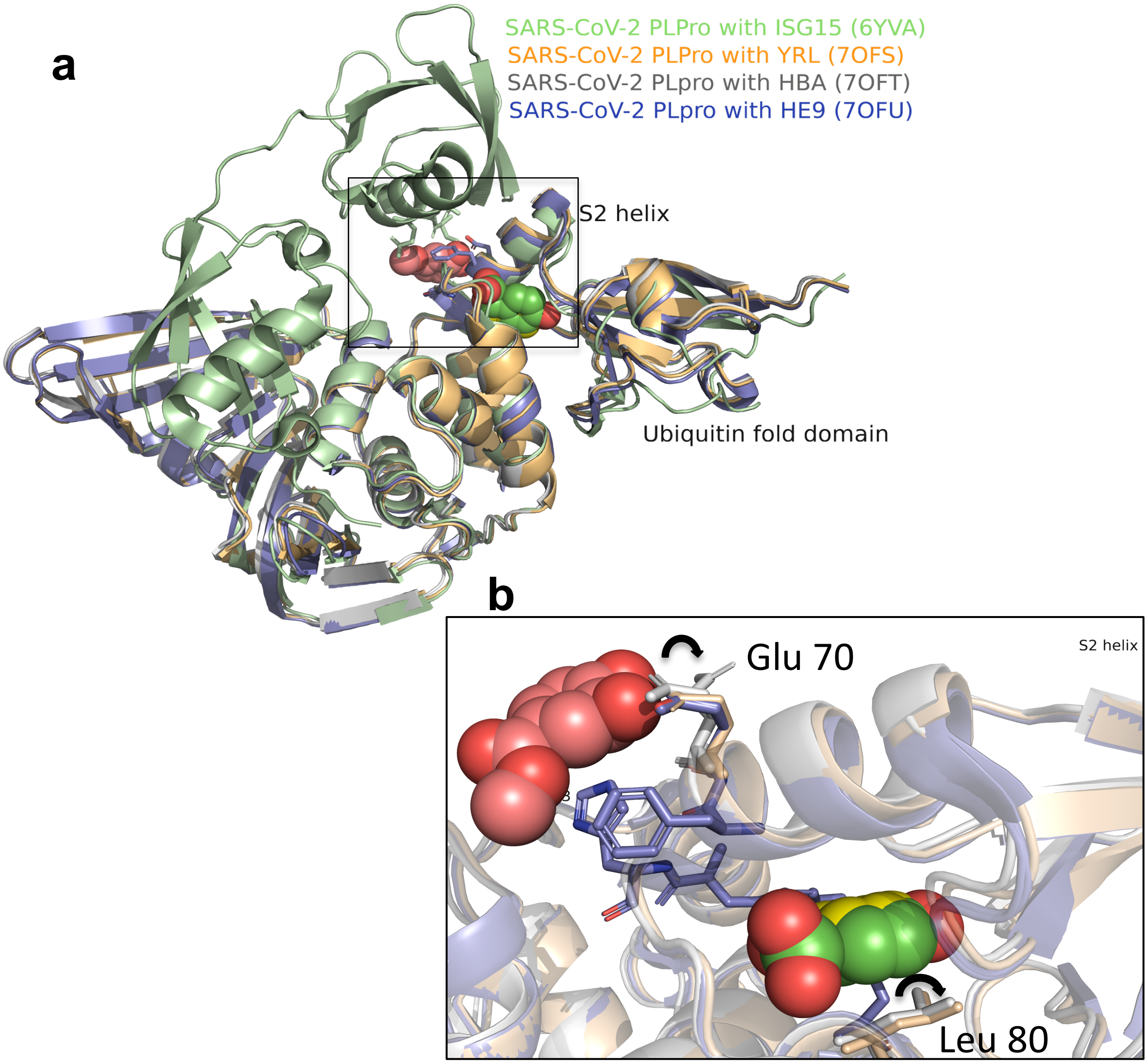

Figure S13.

Conformational changes in PLpro by the binding of the inhibitor compounds

a. Superposition of the crystal structures of SARS-CoV-2 PLpro-C111S in complex with mouse-ISG15 (PDB code 6YVA, in light green) with SARS-CoV-2 PLpro+YRL (PDB code 7OFS, in light orange), SARS-CoV-2 PLpro+HBA (PDB code 7OFT, in grey) and SARS-CoV-2 PLpro+HE9 (PDB code 7OFU, in slate blue). It can be clearly seen that the Ubiquitin-fold domain and the BL2 loop regions are the most dynamic regions in the PLpro molecule.
b. Close-up view of the ISG15 binding site. The residue Leu 80 (sticks in slate blue, apo structure) has to rotate in the complex structures to accommodate the inhibitor molecule. Glu 70 adopts a different side chain conformation when 4-hydroxybenzaldehyde (HBA) interacts with PLpro.

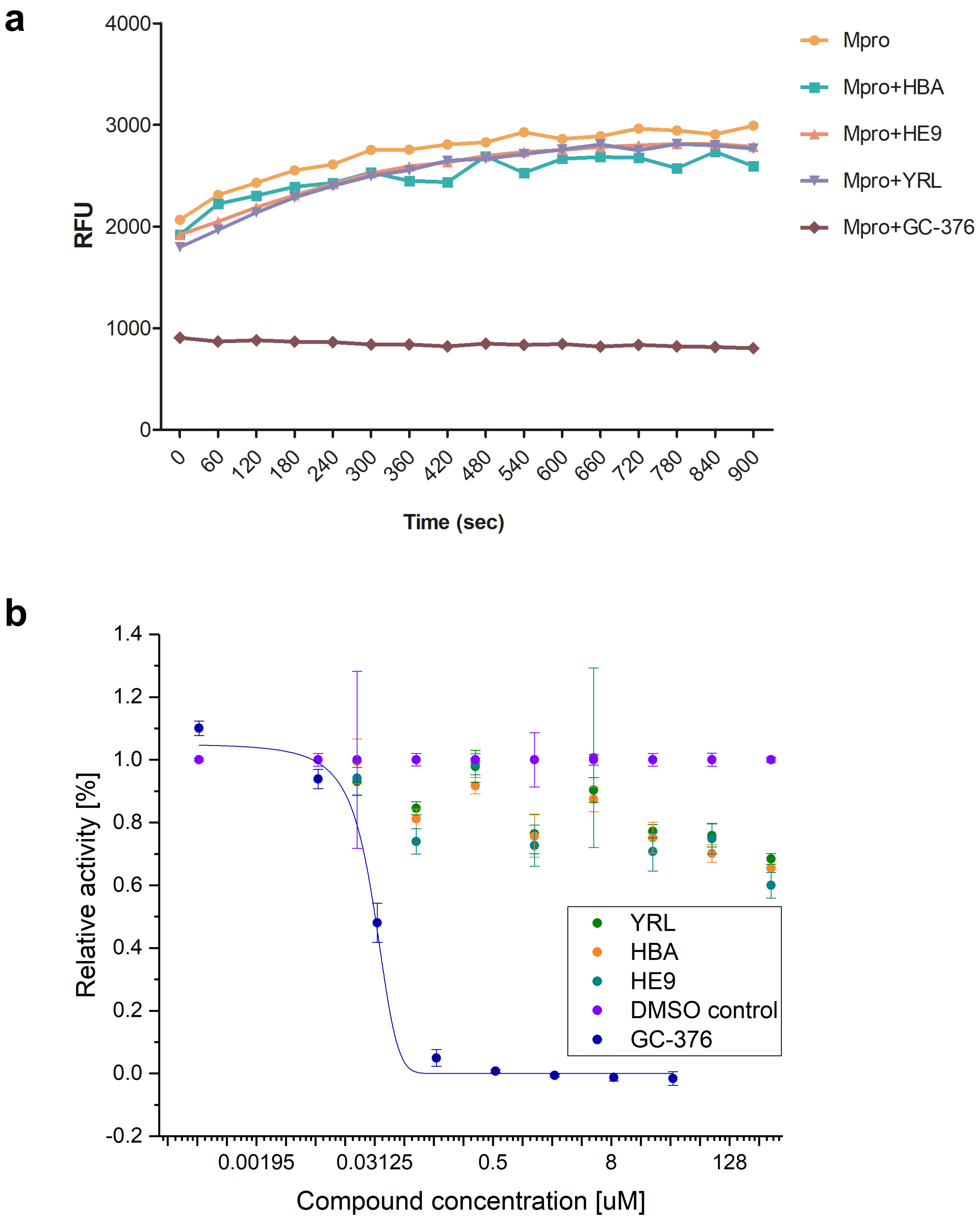
a
4000
3000
2000
1000
0
RFU
0
60
120
180
240
300
360
420
480
540
600
660
720
780
840
900
Time (sec)
Mpro
Mpro+HBA
Mpro+HE9
Mpro+YRL
Mpro+GC-376
b
1.4
1.2
1.0
0.8
0.6
0.4
0.2
0.0
-0.2
Relative activity [%]
0.00195
0.03125
0.5
8
128
Compound concentration [uM]
YRL
HBA
HE9
DMSO control
GC-376

Figure S14.

a. Inhibition assay experiment of the SARS-CoV-2 Main protease (Mpro) applying the three natural compounds in a FRET based activity assay and utilizing 2-AbzSAVLQSGTyr(3-NO2)R-OH as substrate. The compounds were incubated with Mpro for 6h at 10ºC and fluorescence was measured for 15 min with read-out time of 1 min. The known inhibitor GC-376 was used as the positive control.

b. IC50 determination was performed with 2-AbzSAVLQSGTyr(3-NO2)R-OH as the substrate. A gradient concentration of all three compounds YRL, HBA, HE9, a negative DMSO control, and the known inhibitor GC-376 as positive control, in a concentration range from micromolar to nanomolar values. The compounds were incubated with the Mpro for 30 minutes. The IC50 values were calculated by the dose-response-inhibition function after the normalization of the enzymatic activity values with the negative control. The IC50 value for the control compound GC-376 is 1.1 µM and is in agreement with the published data [6]. Individual data points represent mean ± SD from triplicates.

A

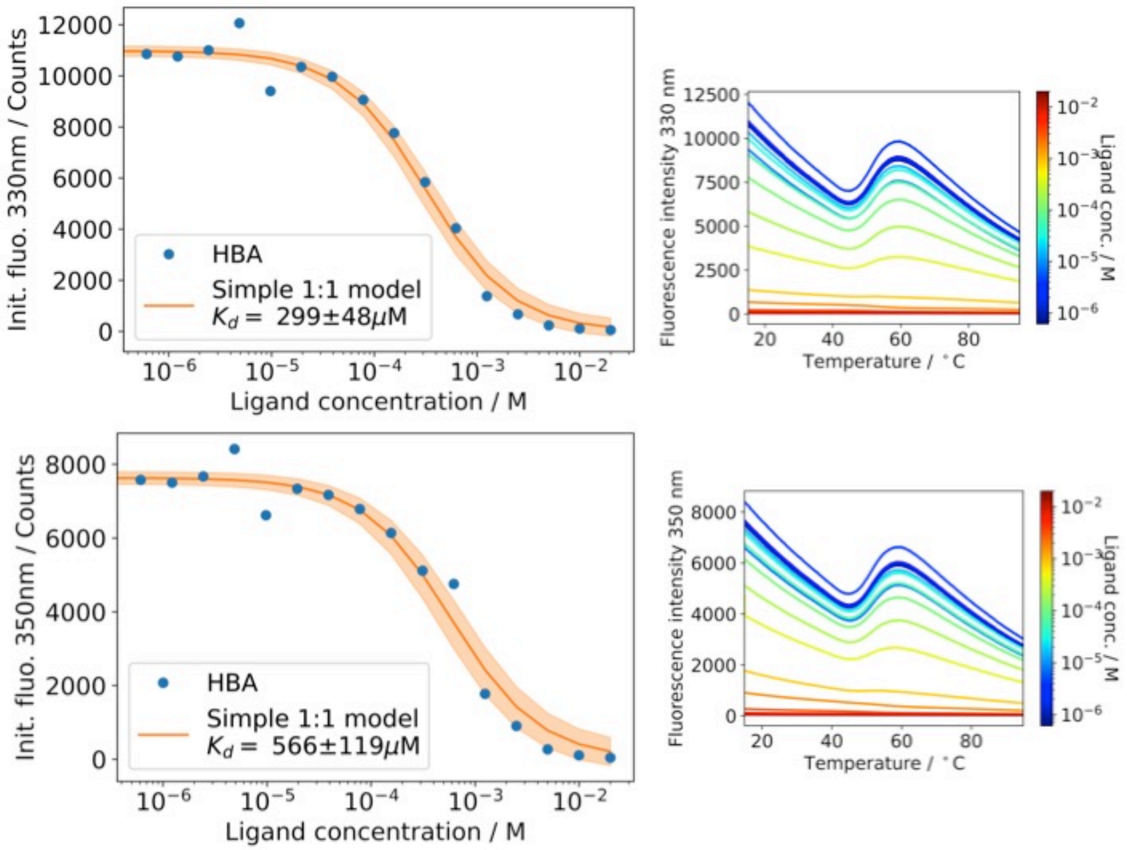

Init. fluo. 330nm / Counts
HBA
Simple 1:1 model
K_d = 299±48μM
Ligand concentration / M
Fluorescence intensity 330 nm
Temperature / °C
Ligand conc. / M
Init. fluo. 350nm / Counts
HBA
Simple 1:1 model
K_d = 566±119μM
Ligand concentration / M
Fluorescence intensity 350 nm
Temperature / °C
Ligand conc. / M

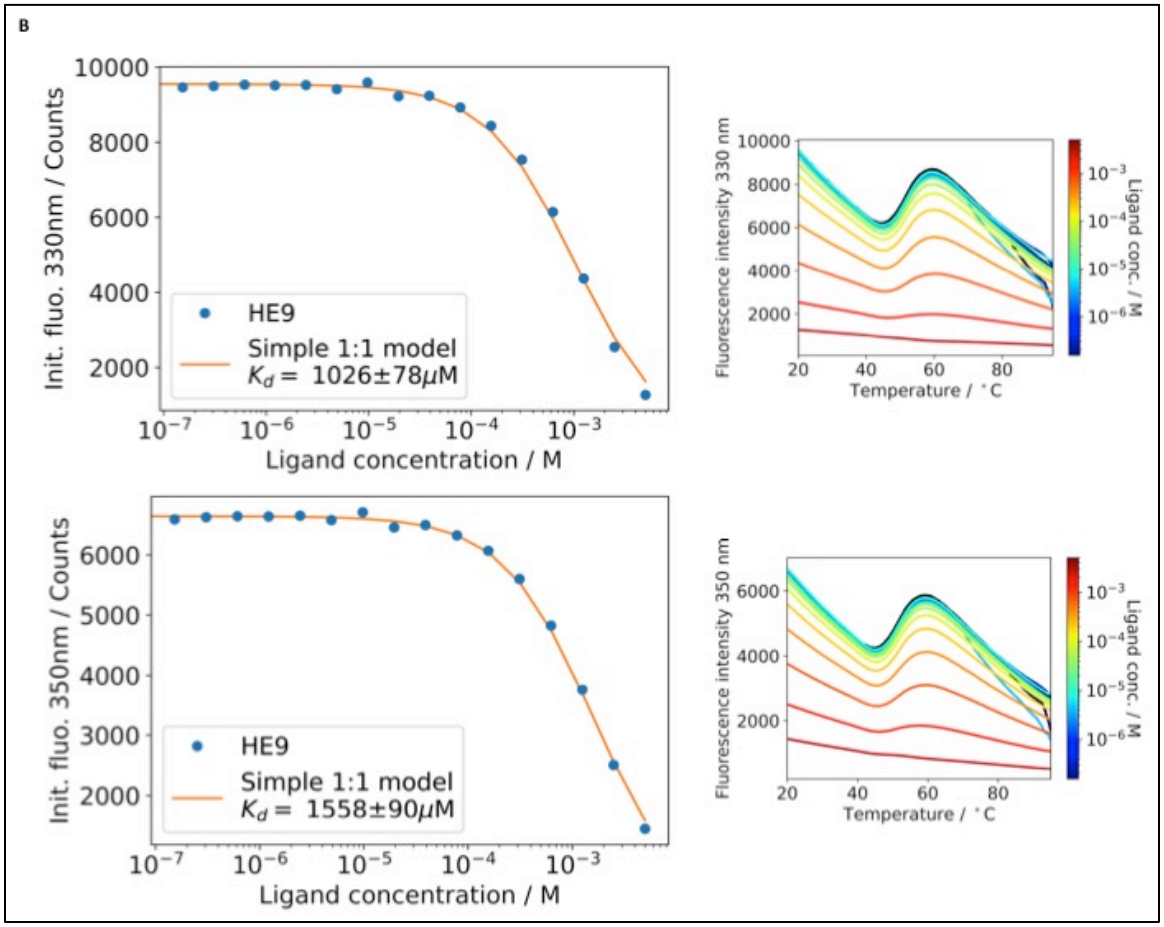

Figure S15.
Fluorescence titration signal (nDSF) for PLpro binding studies at 330nm and 350 nm wavelength in the presence of the inhibitors HBA (A) and HE9 (B).

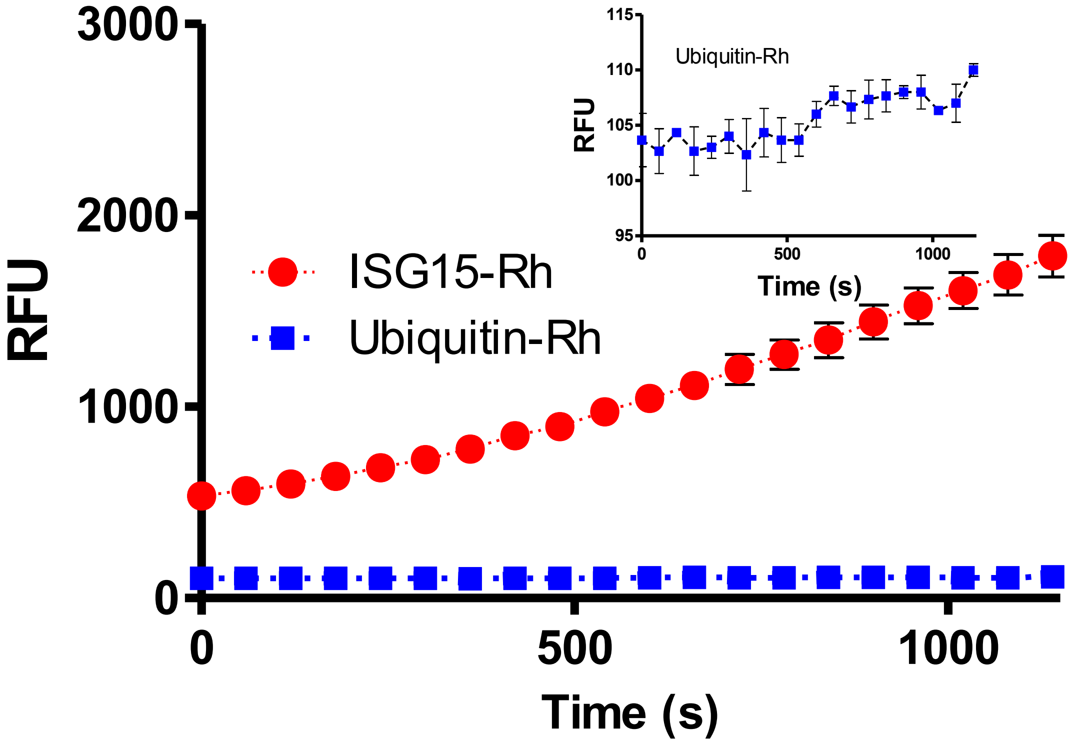

Figure S16.

PLpro enzymatic activity utilizing ISG15-Rh and Ubiquitin-Rh as substrates

PLpro at a concentration of 10nM and ISG15-Rh, Ubiquitin-Rh at a concentration of 100nM were used as substrates. Measured fluorescence values were blank corrected with buffer containing substrates and were measured in triplicates for 60 min with one read-out per minute. The inset shows a enlarged view of the relative fluorescence units with ubiquitin-Rh as substrate. PLpro activity can be clearly seen with ISG15-Rh as substrate and the activity is not significant applying ubiquitin-Rh as substrate.

Supplementary References:

1 Yoshioka, T., Inokuchi, T., Fujioka, S. & Kimura, Y. Phenolic Compounds and Flavonoids as Plant Growth Regulators from Fruit and Leaf of Vitex rotundifolia. *Zeitschrift für Naturforschung C* **59**, 509-514, doi:doi:10.1515/znc-2004-7-810 (2004).

2 Linstrom, P. J., Mallard, W. G., National Institute of, S. & Technology. NIST chemistry webbook. (1997).

3 Wilairat, R. *et al.* Constituents of Schisandra verruculosa. and Their Cytotoxic Effect on Human Cancer Cell Lines. *Pharmaceutical Biology* **44**, 411-415, doi:10.1080/13880200600794105 (2006).

4 Robert, X. & Gouet, P. Deciphering key features in protein structures with the new ENDscript server. *Nucleic Acids Research* **42**, W320-W324, doi:10.1093/nar/gku316 (2014).

5 Günther, S. *et al.* X-ray screening identifies active site and allosteric inhibitors of SARS-CoV-2 main protease. *Science* **372**, 642-646 (2021).

6 Dražić, T., Kühl, N., Leuthold, M. M., Behnam, M. A. M. & Klein, C. D. Efficiency Improvements and Discovery of New Substrates for a SARS-CoV-2 Main Protease FRET Assay. *SLAS discovery : advancing life sciences R & D* **26**, 1189-1199, doi:10.1177/24725552211020681 (2021).
